# Risk Factors for School Absenteeism and Dropout: A Meta-Analytic Review

**DOI:** 10.1007/s10964-019-01072-5

**Published:** 2019-07-15

**Authors:** Jeanne Gubbels, Claudia E. van der Put, Mark Assink

**Affiliations:** Research Institute of Child Development and Education, University of Amsterdam, P.O. Box 15780, Nieuwe Achtergracht 127, 1018 WS Amsterdam, The Netherlands

**Keywords:** Meta-analysis, School absenteeism, Dropout, Risk factor, Risk domain

## Abstract

School absenteeism and dropout are associated with many different life-course problems. To reduce the risk for these problems it is important to gain insight into risk factors for both school absenteeism and permanent school dropout. Until now, no quantitative overview of these risk factors and their effects was available. Therefore, this study was aimed at synthesizing the available evidence on risk factors for school absenteeism and dropout. In total, 75 studies were included that reported on 781 potential risk factors for school absenteeism and 635 potential risk factors for dropout. The risk factors were classified into 44 risk domains for school absenteeism and 42 risk domains for dropout. The results of a series of three-level meta-analyses yielded a significant mean effect for 28 school absenteeism risk domains and 23 dropout risk domains. For school absenteeism, 12 risk domains were found with large effects, including having a negative attitude towards school, substance abuse, externalizing and internalizing problems of the juvenile, and a low parent-school involvement. For dropout, the risk domains having a history of grade retention, having a low IQ or experiencing learning difficulties, and a low academic achievement showed large effects. The findings of the current study contribute to the fundamental knowledge of the etiology of school absenteeism and dropout which in turn contributes to a better understanding of the problematic development of adolescents. Further, more insight into the strength of effects of risk factors on school absenteeism and dropout is important for the development and improvement of both assessment, prevention and intervention strategies.

## Introduction

Problematic school absenteeism is associated with many different life-course problems, such as risky sexual behavior, teenage pregnancy, psychiatric disorders, externalizing behavior, delinquency, and the abuse of alcohol, tobacco, marijuana, and other substances (see, for example, Chou et al. 2006; Egger et al. 2003; Jaafar et al. 2013). In addition, youth showing excessive absenteeism are at high risk for permanent dropout from school (Kearney 2008a), which may lead to economic deprivation and different mental, social, occupational, and marital problems in adulthood (Kogan et al. 2005; Tramontina et al. 2001). To reduce the risk for these problems, it is important to gain insight into risk factors for both problematic school absenteeism (i.e., temporary periods of unexcused school absence) and permanent school dropout. School absenteeism in youth refers to excused or unexcused absences from elementary or secondary (middle/high) school (Kearney 2008a). Whereas excused absenteeism (e.g., absences related to medical illness or injury) could be viewed as non-problematic, unexcused and excessive absenteeism is a problem of serious concern that affects many school systems around the world. Absenteeism rates differ depending on the definition and measurement period. According to the National Center for Education Statistics (2018), 13% of the 8^th^ graders, 14% of the 10^th^ graders, and 15% of the 12^th^ graders were absent at least three days a month, and 6, 5, and 6% were absent at least five days a month, respectively. Until now, many studies on risk factors for school absenteeism and dropout have been performed, but no clear overview of risk factors and their effects was available. The aim of the present study was to provide such an overview by statistically summarizing effects of risk factors by conducting a series of meta-analyses.

Problematic school absenteeism (from now on referred to as school absenteeism) does not refer to a single concept, but to various concepts, including school refusal (absenteeism due to the child’s emotional distress, especially anxiety and depression; King and Bernstein 2001), school phobia (fear-based absenteeism; Tyrrell 2005), truancy (unexcused, illegal, non-anxiety-based absenteeism, which is often linked to a lack of parental monitoring, delinquency, academic problems, or social conditions such as homelessness or poverty; Fremont 2003) and absence from specific lessons. In their interdisciplinary model of school absenteeism, Kearney (2008a) argue that these concepts of school absenteeism are influenced by multiple child, parent, family, peer, school, and community factors. They argue that school absenteeism cases are caused by multiple factors and that the key influential factors are interrelated (e.g., child and parent psychopathology). They also argue that school absenteeism can deteriorate over time from acute, but relatively harmless and occasional absenteeism into regular, and even permanent absenteeism in the form of dropping out of school. This view on how school absenteeism and dropout evolve is in line with the ecological perspective on child development of Bronfenbrenner (1979, 1986). In his influential ecological model, Bronfenbrenner noted that the child interacts with different social ecological systems surrounding the child, such as the family, peers, and the school environment (microsystem), the extended family (exosystem), and the culture, laws, and social-political conditions (macrosystem). In each of these systems, risk factors can be present that increase the risk of negative child behavior, of which school absenteeism is an example. Bronfenbrenner assumed that risk factors in more proximal social systems exert more influence on the child’s development and behavior than risk factors in more distal social systems. Therefore, primary studies aimed at determining risk factors for school absenteeism and school dropout are mainly focused on child-related factors and factors present in the microsystems directly surrounding the child, such as family-, peer-, and school-related factors.

In theoretical models for explaining school absenteeism and dropout such as described above, risk factors play a critical role. Therefore, a large body of research has been directed on identifying risk factors for school absenteeism and school dropout. Some of these risk factors are related to characteristics of the child (e,g., the child’s age [the risk for school absenteeism increases as children become older], internalizing problems, externalizing problems, and a poor physical health), characteristics of the parent (e.g., parental psychiatric problems and parental unemployment), characteristics of the family (e.g. a low socio-economic status and family break-up), characteristics of the school (e.g. large classes, high retention rates, and a poor quality of teachers) or characteristics of the peer group (e.g. antisocial, truant, or delinquent peers). Primary studies examining risk factors for school absenteeism and dropout often show a wide variation in effect size magnitude. Previous reviews of these studies have provided an overview of risk factors or potential causes for school absenteeism (and related concepts) and dropout. Kearney (2008b), for example, reviewed contemporary research on, among other things, the contextual risk factors for school absenteeism and school refusal behavior. Furthermore, Berends and Van Diest (2014) summarized the protective and risk factors for school absenteeism, and King and Bernstein (2001) reviewed studies on problematic family functioning as an important factor contributing to school refusal. However, these reviews were merely qualitative in nature, and until today, the literature on risk factors for school absenteeism and dropout has never been meta-analytically or quantitatively synthesized. In a meta-analysis, the divergent findings of studies on (effects of) risk factors can be summarized to increase insight into whether or not a factor should be designated as a risk factor, and what the true effect of a particular risk factor is. Accordingly, more insight can be gained into all risk factors that play a role in school absenteeism and dropout, leading to a better understanding of the etiology of these problems.

An overview of the variables that are true risk factors for school absenteeism and dropout is also relevant for clinical practice, as this may contribute to the development or improvement of instruments for risk and needs assessment. Risk assessment instruments assess which static (unchangeable in treatment) and dynamic (changeable in treatment) risk factors are present in the environment of a child, and are needed in determining which children should be offered an (preventive) intervention, and with what intensity these children should treated. Needs assessment instruments assess only dynamic risk factors (i.e. the care needs), and are needed in order determining what factors should be targeted in an intervention, so that the risk for school absenteeism or dropout is reduced. Both type of instruments originate from the risk and need principle of the Risk Need Responsivity (RNR) model (Andrews and Bonta 2010; Andrews et al. 1990). This model is used in judicial care as a guidance for offering effective offender assessment and treatment services, and its effectiveness has been proved in several review studies (see, for instance, Andrews et al. 1990; Andrews and Dowden 1999). It can be assumed that this model also applies to problematic and chronic school absenteeism, since criminal recidivism, school absenteeism, and school dropout can all be explained by an accumulation of risk factors in different domains. In addition, there is an overlap between risk factors for school absenteeism and delinquency (Van der Woude et al. 2017).

The present study, then, is important for several reasons. First, examining the effects of different risk factors for school absenteeism and dropout increases the fundamental knowledge of the etiology of these behavioral problems. Second, more insight into the effects of risk factors contributes to the development or improvement of risk and needs assessment instruments. Currently, there are hardly any risk and needs assessment instruments available that assess all relevant risk factors for school absenteeism and dropout, even though such instruments are required for properly referring at-risk juveniles to the most appropriate interventions for reducing risks. Third, the results of this study can support the development and improvement of interventions aimed at preventing (new occurrences of) school absenteeism or dropout. Information on the magnitude of dynamic risk factor effects is essential for determining which risk factors can best be addressed in these interventions.

## The Current Study

This study aimed to synthesize the available evidence on risk factors for school absenteeism and dropout. Specifically, this study was guided by the research questions (1)“What factors can be designated as risk factors for school absenteeism and what is their impact?” and (2) “What factors can be designated as risk factors for school dropout and what is their impact?”. In answering these questions, each (potential) risk factor that was examined in a primary studies was classified into a risk domain, which is as a (broad) group of risk factors that are similar in nature. Next, an overall mean effect was estimated for each of these risk domains in a separate meta-analysis. Finally, as previous literature showed large gender differences in motives for school absenteeism and school dropout (e.g., De Baat and Foolen 2012; Teasley 2004), it was assumed that (effects of) risk factors do not need to be equal for boys and girls. Therefore, this study aimed to answer the following additional research question: (3) “How are risk factor effects influenced by gender?”. To address this final question, the percentage of boys in primary study samples was tested as moderator of the overall effect of each risk domain.

## Method

### Inclusion and Exclusion Criteria

To select relevant studies, several inclusion and exclusion criteria were formulated. First, studies had to examine the effect of at least one (potential) risk factor for school absenteeism and/or dropout. In the current meta-analysis, school absenteeism refers to problematic school absenteeism, which was defined as unexcused absences from school (Kearney 2008a). As described in the Introduction, problematic school absenteeism refers to various concepts, including missing or skipping classes, school non-attendance, and school refusal. Therefore, primary studies reporting on problematic school absenteeism and/or on one or more of these individual concepts were all included. Studies reporting on permitted or excused school absence were not included. School dropout was defined as leaving school prior to earning a high school credential (Kearney 2008b).

Second, only studies examining school absenteeism and/or dropout in primary schools (kindergarten and elementary schools) and secondary schools (middle schools, junior high schools, and high schools) were included. Studies examining absence from college or other forms of post-secondary education were excluded.

Third, as risk factors must precede an outcome (Kraemer et al. 1997), only effect sizes of (potential) risk factors that were present prior to the school absenteeism or school dropout were included. Specifically, primary studies had to report on at least one association between school absenteeism or school dropout and a factor preceding these events, or a factor of which reasonably could be assumed to precede the absenteeism or school dropout based on information described in the primary study. Studies with a longitudinal research design (in which subjects were followed over time) as well as cross-sectional studies (in which subjects were examined at a single point in time) were included. However, factors reported in cross-sectional studies were only included if the factors were already present prior to any (potential) school absenteeism or dropout. This third criterion was to ensure that antecedents of school absenteeism were examined instead of consequences.

Fourth, studies had to report on (1) a measure of bivariate association between a factor and school absenteeism or dropout (e.g., a correlation coefficient) or (2) sufficient information for calculating such an association.

Fifth, given that risk factors for school absenteeism and dropout may be very different in prevalence and nature across cultural settings, only studies that were performed in Western countries were included (i.e., European countries, Australia, New Zealand, Canada, and the US). All primary studies had to be written in Dutch and English to be included.

Sixth, only studies published in peer-reviewed scientific journals or dissertations accessible to the authors of this review were included. Published studies have survived some form of a refereeing and editing process (Dunkin 1996), and although dissertations are not peer-reviewed, they have been evaluated by supervising committees and therefore controlled for quality at least to some extent. As this is not the case for unpublished studies, and as unpublished studies are far more difficult to locate, only published studies and dissertations were searched for and included.

Finally, the aim was not to perform a meta-analysis of the effects of treatment or preventive strategies for reducing school absenteeism and dropout, and because treatment effects may influence risk factor effects, no effects of potential risk factors that are reported in studies examining treatment effects were extracted.

### Search Strategy

Until May 2019, multiple electronic databases were searched to identify relevant studies: Google, Google Scholar, ScienceDirect, PsycINFO, Web of Science, and Sociological Abstracts. The following keywords were used: “truan*”, “dropout”, “drop-out”, “school attendan*”, “school non-attendan*”, “school disengage*”, “class-cutting”, “school refus*”, “school absent*”, “risk factor*”, and “correlate*” (the asterisk represents one or more wildcard characters). Keywords related to “risk factors” were combined with keywords related to “school absenteeism” or “dropout”. Further, the reference list of several relevant reviews and reports were screened (e.g., Berends and Van Diest 2014; De Baat and Foolen 2012; Hammond et al. 2007; Kearney 2008b; Teasley 2004) for relevant studies. Finally, the reference sections of the included primary studies were screened.

**Fig. 1:**
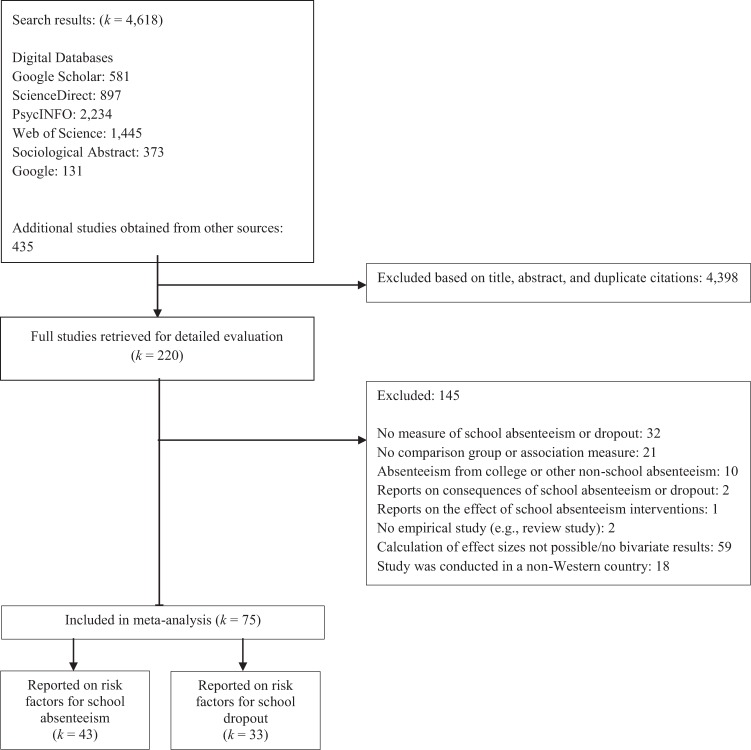
Flow chart of search results

These search methods resulted in 4618 studies. After deduplication and the exclusion of studies based on their title or abstract, 220 studies remained of which the full text was evaluated. Finally, 75 studies met all inclusion criteria and were included in the current study. These studies reported on 71 independent samples. Figure 1 presents a flow chart of the search of studies and Table 1 presents the characteristics of the included studies.

**Table 1:** Characteristics of included studies

Author(s) Pub. year	Focus	*N* ^a^	*n* (absent)^b^	*n* (non-absent)^c^	% Boys^d^	#Risk factors	#Child factors	#Family factors	#School factors	#Peer factors
Alexander et al. 1997	Absenteeism	493–621	194–248	297–373	45.58	31	3	14	14	0
Aloise-Young et al. 2002	Dropout	2203	1213	990	54.23	3	3	0	0	0
Archambault et al. 2009	Absenteeism	11827	–	–	44.60	32	4	0	28	0
Attwood and Croll 2006	Absenteeism	337–344	32–33	304–311	52.51	5	0	2	3	0
Bask and Salmela-Aro 2013	Dropout	878	116	762	–	3	3	0	0	0
Battin-Pearson et al. 2002	Dropout	770	88	682	50.99	34	12	6	12	6
Birkett et al. 2014	Absenteeism	27820–29169	1725–1838	26095–27331	0/100	14	14	0	0	0
Blondal and Adalbjarnardottir 2014	Dropout	835	241	594	46.00	28	2	17	9	0
Blodgett and Lanigan 2018	Absenteeism	2101	270	1831	50.00	1	1	0	0	0
Bobakova et al. 2015	Absenteeism	1380	117	1263	49.70	3	1	0	1	1
Borgna and Struffolino 2017	Dropout	1508–5233	612–759	896–4474	50.50/55.90	3	1	2	0	0
Bosker and Hofman 1994	Dropout	2523	166	2357	–	21	5	5	7	4
Breuner et al. 2004	Absenteeism	233–270	106–123	127–147	38.64	18	17	0	1	0
Bryk and Thum 1989	Absenteeism/Dropout	160–4450	–	–	–	52	10	2	40	0
Burton et al. 2014	Absenteeism	108	29	79	29.00	3	3	0	0	0
Christle et al. 2007	Dropout	196	–	–	50.90	7	2	1	4	0
Claes et al. 2009	Absenteeism	810–3470	513	297–2957	48.40	26	0	14	12	0
(2) Torney-Purta et al 2001										
Corville-Smith et al. 1998	Absenteeism	54	27	27	29.63	11	3	5	2	1
Cratty 2012	Dropout	68401	13188	55213	48.94	18	8	4	6	0
Duncan et al. 2017	Absenteeism	3112	338	2775	50.50	9	6	3	0	0
Dupéré et al. 2017	Dropout	362	183	179	52.29	30	6	8	16	0
Eaton et al. 2008	Absenteeism	2741	1015	1726	43.40	59	57	1	1	0
Echeverria et al. 2014	Absenteeism	93320	5546	87774	50.70	2	2	0	0	0
Egger et al. 2003	Absenteeism	1422	–	–	55.60	11	11	0	0	0
Ensminger et al. 1996	Dropout	1296	291	1005	49.03	9	2	6	1	0
(2) Ensminger and Slusarcick 1992										
Epstein and Sheldon 2002	Absenteeism	12	–	–	–	15	3	6	6	0
Fall and Roberts 2012	Dropout	14781	786	13995	49.40	7	0	1	6	0
Fernandez-Suarez et al. 2016	Dropout	252–264	121–128	131–136	80.74	7	5	2	0	0
Flisher and Chalton 1995	Dropout	400	68	332	50.12	32	30	2	0	0
Fortin et al. 2010	Dropout	102–154	16–50	86–104	0/100	54	10	14	22	8
Gastic 2008	Absenteeism	1578	199–290	1288–1379	53.20	2	0	0	2	0
Gleason and Dynarski 2002	Dropout	2568–2615	154–382	2233–2414	53.00	35	10	10	15	0
(2) Gleason and Dynarski 1994										
Hancock et al. 2018	Absenteeism	5231	544	4687	61.00	23	6	13	0	0
Hagborg et al. 2018	Absenteeism	1147–1240	39–132	1108	49.32	42	10	20	12	0
Henry 2007	Absenteeism	5429–5684	597–890	4539–5087	47.30/48.50	50	16	20	14	0
Hickman et al. 2008	Dropout	119	59	60	50.80	28	3	0	25	0
Hughes et al. 2018	Dropout	735	–	–	52.70	1	0	0	1	0
Hunt and Hopko 2009	Absenteeism	367	255	112	42.00	30	15	13	1	1
Hysing et al. 2015	Absenteeism	8347	–	–	46.50	11	11	0	0	0
Ingul and Nordahl 2013	Absenteeism	94–716	21–80	73–636	47.70	27	19	3	2	3
Ingul et al. 2012	Absenteeism	308–610	101–103	207–507	47.70	46	29	10	6	2
Janosz et al. 1997	Dropout	791	172–335	456–619	46.40/55.37	92	44	24	12	12
Jenkins 1995	Absenteeism	754	–	–	50.00	13	4	7	2	0
Jimerson et al. 2000	Dropout	143	43	100	55.24	17	3	6	5	3
Keppens and Spruyt 2015	Absenteeism	3884	198	3686	–	3	2	0	1	0
Lloyd 1978	Dropout	774–788	143–196	592–631	0/100	36	2	10	24	0
Lounsbury et al. 2004	Absenteeism	248–321	–	–	46.00/48.00/53.00	27	27	0	0	0
Mounteney et al. 2010	Absenteeism	3809	107	3702	51.06	28	23	5	0	0
Morrow and Villodas 2017	Dropout	728	–	–	44.00	6	4	0	0	2
Mullvain 2017	Absenteeism	49	–	–	–	4	1	1	2	0
Peguero et al 2016	Dropout	2550–5950	306–714	2244–5236	48.00/50.00	12	12	0	0	0
Quiroga et al. 2012	Dropout	453	308	145	52.50	11	4	1	5	1
Ramberg et al. 2019	Absenteeism	4956	1160	3296	47.10	3	0	0	3	0
Ramirez et al. 2012	Absenteeism	27110	10163	16947	48.38	10	8	1	1	0
Reid 1984	Absenteeism	154	77	77	–	36	10	6	20	0
(2) Reid 1981										
Rousseau-Salvador et al. 2014	Absenteeism	37–167	12–61	25–106	45,58	8	8	0	0	0
Rumberger 1995	Dropout	17242	–	–	50.00	34	12	10	10	2
Rumberger et al. 1990	Dropout	96–114	48–66	48	48.20	20	10	4	6	0
Sälzer et al. 2012	Absenteeism	3491	–	–	50.00	9	2	1	6	0
Sapharas et al. 2016	Dropout	1689–1761	158–288	1473–1531	0/100	4	0	4	0	0
Schwartz et al. 2009	Absenteeism	40	–	–	37.50	21	17	1	3	0
Sommer 1985	Absenteeism	18–50	9–25	9–25	0/64.00/100	16	2	7	7	0
Steinhausen et al. 2008	Absenteeism	89–244	41–154	48–90	–	54	30	12	8	4
Sznitman et al. 2017	Dropout	3604–13262	901–3846	2703–9416	49.68/51.39	12	8	4	0	0
Taylor 2009	Absenteeism	131	–	–	–	2	0	0	2	0
Teuscher and Makarova 2018	Absenteeism	220	–	–	52.10	7	3	1	2	1
Trampush et al. 2009	Dropout	44–49	24–32	17–20	91.40	10	2	4	4	0
Vaughn et al. 2013	Absenteeism	17125–18443	376–1694	16749	51.00	66	40	14	12	0
Veenstra et al. 2010	Absenteeism	1675	109	1566	–	9	3	4	1	1
Vitaro et al. 2001	Dropout	751	134	617	100	7	1	3	1	2
Zhang et al. 2007	Absenteeism	4745–12464	588–1725	4157–10739	64.75	5	3	1	1	0

*Pub. year* publication year, *Focus* the focus of the study (school absenteeism or dropout), *N* total sample size, *n(absent)* number of school absentees or dropouts in the sample, *n(non-absent)* number of non-absentees or non-dropouts in the sample, % *Boys* percentage of boys in the sample, #*Risk factors* total number of risk factors that were retrieved from the study, #*Child factors* number of child factors that were retrieved from the study, #*Family factors* number of family factors that were retrieved from the study, #*School factors* number of school factors that were retrieved from the study, #*Peer factors* number of peer factors that were retrieved from the study, (2) A primary study using the same sample as the study mentioned directly above, *Absenteeism/Dropout* both school absenteeism and dropout were assessed in study

^a^If more than one sample size was reported in a study (varied by effect size), the range was displayed

^b^If more than one number of truants or dropouts was reported in a study (varied by effect size), the range was displayed

^c^If more than one number of non-truants or non-dropouts was reported in a study (varied by effect size), the range was displayed

^d^If more than one percentage of boys was reported in a study (due to multiple included samples; varied by effect size), all the percentages were displayed, separated by a ‘/’

### Study Coding

Following the guidelines proposed by Lipsey and Wilson (2001), a coding form was developed to code all included primary studies. The primary interest was to synthesize all effects of risk factors that were similar in nature. Across all effect sizes that could be extracted from all included studies, there were too many risk factors to examine individually. For valid and intelligible analyses, each individual risk factor was classified into risk domains, which can be defined as categories of risk factors that are (more or less) similar in nature. According to the interdisciplinary model of school absenteeism of Kearney (2008a) and the ecological model of Bronfenbrenner (1979; 1986; see Introduction), these risk domains are related to (1) characteristics of the child; (2) characteristics of parents or caretakers, and the family; (3) characteristics of the school; or (4) characteristics of peer relationships and interactions with peers. For each extracted risk factor, it was first determined whether the factor was related to the child, the family, the school, or the peers. Next, a risk factor was further classified into more specific risk domains, and this procedure was done separately for school absenteeism and dropout. The online Appendix A shows an overview of the risk domains that were examined in this review. In the end, all risk factors for school absenteeism were classified into one of 44 mutually exclusive risk domains, of which 24 were related to child characteristics, 11 to family characteristics, 6 to school characteristics, and 3 to peer and peer-interaction characteristics. Risk factors for school dropout were classified into 42 mutually exclusive risk domains, of which 23 were related to child characteristics, 12 to family characteristics, 4 to school characteristics, and 3 to peer and peer interaction characteristics.

For descriptive purposes, several sample and study characteristics were coded. However, it was decided to only examine the moderating effect of one sample characteristic, namely the percentage of boys within the sample. This variable was tested as a moderator, as it is known that there can be large gender differences in motives for school absenteeism and dropout (e.g., De Baat and Foolen 2012; Teasley 2004). In coding studies for meta-analytic research, it is common practice to retrieve a large amount of information from primary studies (see for instance, Cooper 2010; Lipsey and Wilson 2001), after which the moderating effect of a variety of study, sample, and research design descriptors is tested. However, since the problem of multiple testing often dealt with in primary studies (e.g., Tabachnik and Fidell 2013) is equally present in meta-analytic research, it was decided to only test the variable that seemed most relevant in light of the aims of the present review. Further, in order to gain sufficient statistical power in the moderator analyses, the variable percentage of boys in the sample was only tested as a moderator when this variable was based on at least five studies. The other coded variables did not meet this criterion, which was also reason not to test any other variable as a moderator within the risk domains.

In coding all included studies, two coding rounds were completed. First, 10 studies that were eligible for inclusion (7 school absenteeism studies and 3 dropout studies, reporting on a total of 282 risk factors) were randomly selected and coded by the first author and an and an independent assistant researcher. Next, the independent codings were compared and percentages of agreement were calculated. A perfect agreement (100%) was found for the percentage of boys in the sample, and the number of extracted effect sizes from each primary study. The agreement for the double-coded effect sizes was calculated by dividing the number of matching codings (268) by the total number of double-coded effect sizes (282), which was 95%. All discrepancies in the 5% non-matching effect size codings were discussed by the two coders until full consensus was reached. In the second coding round, the first author coded the remaining 65 studies. Finally, the classification of every extracted (potential) risk factor into risk domains was discussed by the first, second, and third author of this study. Therefore, the interrater agreement for the risk domain variable was perfect (100%).

## Calculation of Effect Sizes and Statistical Analyses

In this review, the correlation coefficient (*r*) was chosen as common effect size for risk factor effects, meaning that a correlation was calculated for each extracted (potential) risk factor. The correlations were directly obtained from the included studies, or calculated using information that was reported in the studies (such as proportions, means and standard deviations, odds-ratio’s, or *F* or *t* values). In these calculations, the formulas of Ferguson (1966), Rosenthal (1994), and Lipsey and Wilson (2001) were used. A positive *r* value was assigned to a factor that was *more* present in youth showing school absenteeism or dropout than in youth not showing these problems, whereas a negative *r* value was assigned to a factor that was *less* present in youth showing school absenteeism or dropout. If a risk factor effect was reported as non-significant in primary studies without further statistical information to calculate the actual effect size, an effect size of zero was assigned to the factor (see also Durlak and Lipsey 1991). This procedure was applied to one study, in which two factors were described as non-significant. After all correlation coefficients were obtained, the *r* values were transformed into Fisher's *z* values, as correlations are non-normally distributed (see, for instance, Lipsey and Wilson 2001).

Because most studies reported on more than one risk factor for school absenteeism or dropout, a traditional random effects (two-level) model was extended to a three-level random effects model (Cheung 2014; Houben et al. 2015; Van den Noortgate et al. 2013, 2014). A major advantage of this three-level approach to meta-analysis is that all relevant effects reported in each primary study can be included, implying that all relevant information is preserved. As a result, there no information is lost and (moderator) effects can be estimated more precisely and with maximum power in the statistical analyses (Assink and Wibbelink 2016). In a three-level random effects meta-analytic model, three sources of variance are taken into account: sampling variance of the observed effect sizes (Level 1), variance between effect sizes extracted from the same study (Level 2), and variance between studies (Level 3). In an intercept-only model, the intercept represents the estimate of the overall or mean effect of a single risk domain. If variation in effect sizes extracted from the same study (i.e., level 2 variance) and/or variation in effect sizes extracted from different studies (i.e., level 3 variance) was significant, the model was extended with the potential moderating variable percentage of boys to determine whether this variable can explain any significant variance. In a number of included studies, variables were examined as risk factors using the same sample. As this induces dependency in effect sizes that are extracted from these studies, the same study identification number was given to these studies, so that effect size dependency is accounted for.

In the statistical environment R (version 3.5.1; R Core Team 2015), the function “rma.mv” of the metafor-package (Viechtbauer 2010) was used to conduct the statistical analyses. The R syntaxes were written so that the three sources of variance were modeled (Assink and Wibbelink 2016). In testing individual regression coefficients and calculating corresponding confidence intervals, a *t*-distribution was used (Knapp and Hartung 2003). To determine the significance of the level 2 and level 3 variance, the full model was compared to a model excluding one of these variance parameters in two separate log-likelihood ratio tests. If significant level-2 and/or level-3 variance was detected, the distribution of effect sizes was considered to be heterogeneous. This indicated that effect sizes could not be treated as estimates of one common effect size, meaning that moderator analyses could be performed. All model parameters were estimated using the restricted maximum likelihood estimation method. Prior to the analyses, a dichotomous dummy variable was created for each category of a discrete variable and continuous variables were centered around their mean. The log-likelihood-ratio-tests were performed one-tailed and all other tests were performed two-tailed. A *p*-value < 0.05 was considered as statistically significant. Finally, it should be noted that all significant and non-significant results of all performed analyses are reported. No significant or non-significant result of any analysis was left out.

### Assessment of Bias

Despite an extensive search for studies on risk factors for school absenteeism and dropout, it is possible that relevant studies were missed due to limitations in the search strategy or different forms of bias, such as publication bias or subjective reporting bias. To examine whether (a form of) bias was present in the estimated overall effects of risk domains, three analyses were conducted that are all three based on the association between effect size and sample size that is expected when bias is present in the effect sizes that are to be synthesized. First, a funnel-plot-based trim and fill method was conducted (Duval and Tweedie 2000a, 2000b). This means that in case of an asymmetrical distribution of effect sizes (i.e., an asymmetrical funnel plot), the symmetry of the distribution is restored by imputing effect size estimates from “missing” studies. Effect sizes imputed to the left of the estimated mean effect imply that below average effect sizes were underrepresented and that the estimated mean effect may be an overestimation of the true effect. On the other hand, imputation of effect sizes to the right of the estimated mean effect indicates that above average effect sizes were underrepresented and that the estimated mean effect may be an underestimation of the true effect. Second, a three-level funnel plot test was conducted in which effect sizes were regressed on the sample sizes in a 3-level meta-analytic model, in which effect size dependency is accounted for. In this model, a significant slope is an indication of bias. Third, an adapted Egger”s test was conducted in which effect sizes were regressed on standard errors in a 3-level meta-analytic model. In this test, effect size dependency was also accounted for and a significant slope is once again an indication of bias. These bias assessment analyses were also performed in the R environment (Version 3.5.1; R Core Team 2015) with the functions “trimfill” and “rma.mv” of the metafor package (Viechtbauer 2010).

## Results

In total, *k* = 75 studies published between 1978 and 2019 were included with *k=* 43 studies reporting on factors for school absenteeism and *k=* 33 studies reporting on factors for dropout. For specifically school absenteeism, 43 studies with 41 non-overlapping samples (*N* = 243,296 pupils) were included, from which 781 effect sizes were extracted. The average percentage of boys in the samples of these studies was 47.9%. All included studies together reported on at least *n* = 26,230 absentees and at least *n* = 189,437 non-absentees. Exact numbers of these groups could not be given, as in some studies the specific number of absentees and non-absentees was not reported. The included studies were conducted in the USA (*k* = 21), Canada (*k* = 3), Australia (*k* = 1), and Europe (*k* = 16).

The 33 studies on school dropout used 31 non-overlapping samples with a total sample size (*N*) of 136,392 pupils. These studies examined at least *n* = 21,625 school dropouts and at least *n* = 95,813 non-dropout (again, some of the dropout studies did not report on the specific number of dropouts and non-dropouts), and reported on 635 effect sizes. The average percentage of boys in the samples of these studies was 51.8%. The dropout studies were conducted in USA (*k* = 21), Canada (*k* = 5), and Europe (*k* = 6).

### Overall Effects of Risk Domains for School Absenteeism

Table 2 presents an estimated overall effect for each of the 44 risk domains for school absenteeism in descending order, separately for child-, family-, school- and peer related risk domains. The overall effects of 28 domains were significant and positive in direction (including 16 child-related risk domains, 9 family-related risk domains, and 3 school-related risk domains), implying that these domains can be regarded as true risk domains for school absenteeism. The magnitude of the effects of these risk domains ranged from small (i.e., *r=* 0.099 for “low IQ/learning difficulties”) to large (i.e., *r=* 0.553 for “having a negative school attitude”) based on the criteria of Rice and Harris (2005) for interpreting effect sizes. Significant large overall effects (*r* ≥ 0.252) were found for 11 risk domains (indicated in Table 2 with “^a^”), including the child related risk domains “having a negative school attitude”, “anti-social behavior/cognitions”, “smoking”, “drug abuse”, “alcohol abuse”, “other internalizing problems”, “psychiatric symptoms or disorders”, and “being a sexual minority”; the family related risk domains “low parental school involvement” and “history of child abuse victimization”; and the school risk domain “poor pupil-teacher relationship”. Further, various risk domains with a significant medium overall effect (0.160 < *r* < 0.252) or a significant small effect (*r* < 0.160) were found (indicated in Table 2 with “^b^” and “^c^”, respectively).

**Table 2:** Overall effect sizes of all risk domains for school absenteeism

Domain of risk factors	# Studies	# ES	Mean *z* (SE)	95% CI	Sig. mean *z* (*p*)	% Var. at level 1	Level 2 variance	% Var. at level 2	Level 3 variance	% Var. at level 3	Mean *r*
Child domains (#24)											
Having a negative school attitude	11	72	0.553 (0.222)	(0.110, 0.996)	<0.001^***^	0.0	0.003^***^	0.6	0.536^***^	99.4	0.503^a^
Anti-social behavior/cognitions	19	53	0.457 (0.180)	(0.096, 0.818)	0.014^*^	0.0	0.018^***^	3.0	0.604^***^	97.0	0.428^a^
Low academic self-concept	3	5	0.418 (0.189)	(−0.105, 0.942)	0.091^+^	1.9	0.033^***^	28.6	0.079	69.6	0.395
Smoking	2	9	0.350 (0.048)	(0.239, 0.461)	<0.001^***^	1.4	0.021^***^	98.6	0.000	0.0	0.336^a^
Drug abuse	7	24	0.340 (0.069)	(0.196, 0.483)	<0.001^***^	0.5	0.011^***^	27.7	0.028^*^	71.8	0.327^a^
Alcohol abuse	7	35	0.322 (0.026)	(0.270, 0.375)	<0.001^***^	1.1	0.023^***^	98.9	0.000	0.0	0.311^a^
Other internalizing problems	11	33	0.317 (0.122)	(0.068, 0.565)	0.014^*^	0.9	0.008^***^	5.0	0.156^***^	94.0	0.307^a^
Psychiatric symptoms/disorders	5	7	0.313 (0.047)	(0.198, 0.428)	<0.001^***^	20.4	0.003	29.2	0.005	50.5	0.303^a^
Being a sexual minority	2	7	0.280 (0.079)	(0.086, 0.474)	0.012^*^	0.3	0.005^***^	36.9	0.008	62.8	0.273^a^
Delinquent behavior	7	20	0.258 (0.139)	(−0.032, 0.549)	0.079^+^	0.1	0.019^***^	13.4	0.123^**^	86.5	0.252
Depression	8	13	0.242 (0.032)	(0.173, 0.311)	<0.001^***^	5.8	0.008^***^	94.2	0.000	0.0	0.237^b^
Low academic achievement	11	22	0.236 (0.049)	(0.135, 0.337)	<0.001^***^	1.0	0.024^***^	72.4	0.009	26.5	0.232^b^
High sexual involvement	2	8	0.233 (0.064)	(0.081, 0.384)	0.008^**^	1.2	0.032^***^	98.8	0.000	0.0	0.229^b^
Showing risky behavior	2	8	0.230 (0.067)	(0.073, 0.388)	0.011^*^	0.9	0.003^***^	28.1	0.008^*^	71.0	0.226^b^
Poor physical health	10	57	0.180 (0.042)	(0.097, 0.264)	<0.001^***^	0.8	0.010^***^	42.5	0.013^***^	56.7	0.178^b^
Risky coping/personality profile	7	34	0.159 (0.020)	(0.117, 0.200)	<0.001^***^	28.9	0.010^***^	71.1	0.000	0.0	0.158^c^
Not being religious	2	3	0.142 (0.040)	(−0.031, 0.316)	0.072^+^	7.4	0.004^***^	92.6	0.000	0.0	0.141
Age (being older)	12	17	0.127 (0.029)	(0.065, 0.189)	<0.001^***^	4.5	0.003^***^	26.8	0.006	68.7	0.126^c^
Anxiety	9	25	0.116 (0.047)	(0.018, 0.214)	0.022^*^	3.5	0.007^***^	33.8	0.013^***^	62.7	0.115^c^
High impact/negative life events	4	8	0.112 (0.062)	(−0.033, 0.258)	0.111	0.9	0.026^***^	99.1	0.000	0.0	0.112
* History of grade retention*	1	1	0.100 (0.017)	(0.068, 0.132)	<0.001^***^	100.0	–	–	–	–	0.100^c^
Low IQ/learning difficulties	5	6	0.099 (0.033)	(0.014, 0.184)	<0.001^***^	5.0	0.005	95.0	0.000	0.0	0.099^c^
Negative or no leisure activities	4	10	0.081 (0.084)	(−0.110, 0.271)	0.364	7.0	0.005^*^	16.2	0.024^*^	76.8	0.081
Having a job	2	6	0.071 (0.096)	(−0.177, 0.319)	0.494	0.5	0.005^***^	21.2	0.017^*^	78.3	0.071
Ethnicity (being non-white)	15	36	0.024 (0.030)	(−0.037, 0.086)	0.431	0.6	0.006^***^	43.4	0.008^+^	56.0	0.024
Family domains (#11)											
Low parental school involvement	4	12	0.279 (0.108)	(0.042, 0.516)	0.025^*^	0.3	0.001^***^	1.2	0.045^***^	98.5	0.272^a^
History of child abuse victimization	4	19	0.263 (0.023)	(0.214, 0.312)	<0.001^***^	8.6	0.009^***^	91.4	0.000	0.0	0.257^a^
* Low attachment to parent*	1	1	0.224 (0.024)	(0.175, 0.264)	<0.001^***^	100.0	–	–	–	–	0.220^b^
Large family size	2	2	0.197 (0.120)	(−1.323, 1.717)	0.348	35.6	0.010	32.2	0.010	32.2	0.194
Family structure (no nuclear family)	11	24	0.189 (0.046)	(0.093, 0.285)	<0.001^***^	0.5	0.050^***^	99.5	0.000	0.0	0.187^b^
Parental mental/physical problems	1	3	0.188 (0.040)	(0.017, 0.360)	0.042^*^	4.0	0.005^***^	96.0	0.000	0.0	0.186^b^
Low parental support/acceptance	4	12	0.184 (0.027)	(0.124, 0.245)	<0.001^***^	10.1	0.005^***^	89.9	0.000	0.0	0.182^b^
Low parental education	6	17	0.156 (0.041)	(0.068, 0.243)	0.002^**^	2.2	0.011^***^	74.6	0.004	23.2	0.155^c^
Ineffective family systems	6	15	0.154 (0.071)	(0.001, 0.306)	0.049^*^	7.1	0.002	6.7	0.024^**^	86.3	0.153^c^
Low family SES	20	50	0.135 (0.047)	(0.042, 0.229)	0.005^**^	0.8	0.010^***^	23.7	0.033^***^	75.4	0.134^c^
Low parental control	6	16	0.124 (0.028)	(0.064, 0.184)	<0.001^***^	3.1	0.004^***^	61.8	0.002	35.1	0.123^c^
Sibling at school	1	2	0.065 (0.035)	(−0.382, 0.512)	0.315	53.8	0.001	46.2	0.000	0.0	0.065
* Parental absenteeism in past*	1	1	0.000 (0.200)	(−0.373, 0.373)	1.000	100.0	–	–	–	–	0.000
School domains (#6)											
Distance to school (short)	1	3	0.518 (0.228)	(−0.464, 1.500)	0.151	71.1	0.045	28.9	0.000	0.0	0.476
Poor pupil-teacher relationship	6	9	0.294 (0.057)	(0.163, 0.425)	<0.001^***^	1.6	0.007^***^	33.9	0.013	64.5	0.286^a^
education/education	6	21	0.233 (0.094)	(0.037, 0.429)	0.022^*^	1.6	0.007^***^	13.2	0.047^***^	85.2	0.229^b^
Negative school/class climate	8	26	0.185 (0.028)	(0.128, 0.242)	<0.001^***^	3.6	0.018^***^	96.4	0.000	0.0	0.183^b^
Public school (vs. private)	1	2	0.098 (0.062)	(−0.688, 0.885)	0.358	9.9	0.007^**^	90.1	0.000	0.0	0.098
Large classes/schools	3	6	0.044 (0.110)	(−0.240, 0.328)	0.709	10.3	0.000	0.0	0.027^*^	89.7	0.044
Peer domains (#3)											
Having many friends	2	3	0.204 (0.149)	(−0.437, 0.846)	0.304	18.0	0.000	0.0	0.038	82.0	0.201
Poor social competence	7	10	0.106 (0.062)	(−0.034, 0.247)	0.120	9.1	0.032^***^	90.9	0.000	0.0	0.106
* No subculture affiliation*	1	1	0.060 (0.027)	(0.008, 0.112)	0.013^*^	100.0	–	–	–	–	0.060^c^
Being bullied	5	7	0.011 (0.105)	(−0.246, 0.269)	0.919	3.5	0.001	2.9	0.048^*^	93.6	0.011

# *studies* number of studies, # *ES* number of effect sizes, *SE* standard error, *CI* confidence interval, *Sig* significance, *Mean z* mean effect size (Fisher’s *z*), % *Var* percentage of variance explained, *Level* 2 *variance* variance between effect sizes from the same study, *Level* 3 *variance* variance between studies, *Meanr* the correlation coefficient corresponding to the mean effect size *z*

Risk factors that could not be classified into one of the 44 created risk domains for school absenteeism are presented in *italics*

^+^*p* < 0.10; *p < 0.05; **p < 0.01; ***p < 0.001

^a^Significant large effect (according to the guidelines of Rice and Harris 2005)

^b^Significant medium effect (according to the guidelines of Rice and Harris 2005)

^c^Significant small effect (according to the guidelines of Rice and Harris 2005)

For 15 domains, the estimated overall effect did not significantly deviate from zero implying that these domains cannot be regarded as *risk* domains given the present results. Of these 15 domains, three had as trend significant overall effect. Table 2 also shows the effects of 4 single factors (presented in *italics*) that could not be classified in any of the created risk domains, due to their unique nature. The effect of the factors “history of grade retention”, “low attachment to parents”, and “no subculture affiliation” were significant and medium to small in size. The effect of “parental absenteeism in past” was not significant, implying that this variable was not identified as a risk factor for school absenteeism.

### Overall Effects of Risk Domains for Dropout

Table 3 shows the overall effects of the 42 risk domains for school dropout. A significant effect in a positive direction was found for 23 risk domains, including 13 child-related domains, 7 family-related domains, 1 school-related domain, and 2 peer-related domains. Based on the criteria of Rice and Harris (2005), the magnitude of the significant overall effects ranged from small (i.e., *r* = 0.062 for “ethnicity”) to large (i.e., *r* = 0.365 for “history of grade retention”). Three child related risk domains with a large significant effect were found (*r* ≥ 0.299; indicated in Table 3 with “^a^”), including “history of grade retention”, “low IQ/learning difficulties”, and “low academic achievement”. Table 3 also lists several risk domains with a significant medium overall effect (0.192 < *r* < 0.299) or a significant small overall effect (*r* < 0.192) (indicated with “^b^” and “^c^”, respectively).

**Table 3:** Overall effect sizes of all risk domains for school dropout

Domain of risk factors	# Studies	# ES	Mean *z* (SE)	95% CI	Sig. mean *z* (*p*)	% Var. at level 1	Level 2 variance	% Var. at level 2	Level 3 variance	% Var. at level 3	Mean *r*
Child domains (#23)											
History of grade retention	10	18	0.363 (0.051)	(0.257, 0.470)	<0.001^***^	0.3	0.020^***^	63.9	0.011^+^	35.8	0.348^a^
Low IQ/learning difficulties	8	12	0.338 (0.070)	(0.184, 0.491)	<0.001^***^	1.7	0.053^***^	98.3	0.000	0.0	0.326^a^
Low academic achievement	21	92	0.327 (0.033)	(0.260, 0.393)	<0.001^***^	2.7	0.022^***^	60.5	0.013^**^	36.8	0.316^a^
Psychiatric symptoms/disorders	2	4	0.276 (0.042)	(0.142, 0.411)	0.007^**^	1.1	0.001^***^	30.9	0.003	68.0	0.269^b^
Drug abuse	5	9	0.252 (0.054)	(0.128, 0.376)	0.002^**^	10.7	0.016^***^	78.2	0.002	11.1	0.247^b^
Anti-social behavior/cognitions	14	35	0.241 (0.038)	(0.163, 0.319)	<0.001^***^	3.0	0.008^***^	32.5	0.015^***^	64.5	0.236^b^
Delinquent behavior	3	10	0.227 (0.027)	(0.167, 0.287)	<0.001^***^	50.1	0.000	12.2	0.001	37.8	0.223^b^
Low academic self-concept	4	8	0.221 (0.101)	(−0.018, 0.461)	0.056^+^	0.8	0.003^+^	7.4	0.038^*^	91.8	0.217
* Poor general well-being*	1	1	0.214 (0.020)	(0.173, 0.247)	<0.001^***^	100.0	–	–	–	–	0.210^b^
Having a negative school attitude	11	50	0.213 (0.028)	(0.157, 0.269)	<0.001^***^	1.0	0.021^***^	85.9	0.003	13.1	0.210^b^
Age (Being older)	4	7	0.198 (0.044)	(0.091, 0.304)	0.004^**^	12.9	0.010^**^	87.1	0.000	0.0	0.195^b^
* Adverse childhood experiences*	1	1	0.187 (0.037)	(0.116, 0.256)	<0.001^***^	100.0	–	–	–	–	0.185^c^
High sexual involvement	3	6	0.172 (0.098)	(−0.079, 0.423)	0.139	2.3	0.056^***^	97.7	0.000	0.0	0.170
Poor physical health	4	7	0.158 (0.045)	(0.048, 0.268)	0.013^*^	1.2	0.014^***^	98.8	0.000	0.0	0.157^c^
Other internalizing problems	4	10	0.141 (0.039)	(0.052, 0.230)	0.006^***^	12.3	0.004^***^	41.7	0.004^+^	45.9	0.140^c^
Smoking	2	3	0.126 (0.295)	(−1.143, 1.394)	0.712	0.4	0.018^***^	10.4	0.159	89.2	0.125
Not being religious	1	2	0.113 (0.025)	(−0.207, 0.433)	0.140	100.0	0.000	0.0	0.000	0.0	0.113
Showing risky behavior	1	14	0.109 (0.040)	(0.023, 0.195)	0.017^*^	11.4	0.020^***^	88.6	0.000	0.0	0.109^c^
Alcohol abuse	2	5	0.101 (0.214)	(−0.495, 0.696)	0.664	3.0	0.001	0.8	0.089^**^	96.3	0.101
Having a job	1	2	0.088 (0.025)	(−0.232, 0.408)	0.178	100.0	0.000	0.0	0.000	0.0	0.088
Negative or no leisure activities	5	11	0.084 (0.047)	(−0.020, 0.188)	0.101	2.6	0.004^***^	35.6	0.007	61.8	0.084
Depression	2	4	0.069 (0.055)	(−0.105, 0.244)	0.296	34.2	0.008^*^	65.8	0.000	0.0	0.069
Ethnicity (being non-white)	13	56	0.062 (0.025)	(0.012, 0.112)	0.017^*^	0.4	0.018^***^	87.2	0.003	12.3	0.062^c^
Risky coping/personality profile	5	20	0.057 (0.034)	(−0.014, 0.129)	0.110	3.7	0.021^***^	96.3	0.000	0.0	0.057
Anxiety	2	3	0.009 (0.022)	(−0.086, 0.105)	0.719	100.0	0.000	0.0	0.000	0.0	0.009
Family domains (#12)											
Low family SES	16	34	0.226 (0.044)	(0.137, 0.316)	<0.001^***^	1.1	0.010^***^	28.0	0.024^***^	70.9	0.222^b^
Low parental education	10	20	0.203 (0.040)	(0.120, 0.286)	<0.001^***^	1.0	0.003^***^	17.7	0.013^***^	81.4	0.200^b^
Large family size	3	4	0.197 (0.042)	(0.065, 0.329)	0.018^*^	18.9	0.000	0.0	0.004^+^	81.1	0.194^b^
Ineffective family systems	3	6	0.182 (0.101)	(−0.078, 0.442)	0.131	3.7	0.024^***^	56.6	0.017	39.6	0.180
Family structure (no nuclear family)	8	18	0.180 (0.024)	(0.130, 0.230)	<0.001^***^	5.1	0.008^***^	94.9	0.000	0.0	0.178^c^
Low parental support/acceptance	8	16	0.178 (0.051)	(0.068, 0.288)	0.004^**^	2.3	0.012^***^	47.8	0.013^+^	49.9	0.176^c^
Poor parent-child relationship	4	9	0.165 (0.116)	(−0.102 0.432)	0.191	5.3	0.002	3.1	0.047^**^	91.6	0.164
* Age of mother (being younger)*	1	1	0.164 (0.043)	(0.081, 0.243)	<0.001^***^	100.0	–	–	–	–	0.163^c^
Low parental school involvement	7	25	0.150 (0.040)	(0.069, 0.232)	<0.001^***^	1.6	0.010^***^	56.8	0.007^*^	41.6	0.149^c^
Low parental control	8	21	0.135 (0.059)	(0.013, 0.258)	0.032^*^	3.2	0.004^***^	13.6	0.023^***^	83.1	0.134^c^
Sibling dropped out	1	2	0.122 (0.054)	(−0.560, 0.804)	0.264	6.7	0.005^***^	93.3	0.000	0.0	0.121
Parental alcohol use	1	2	0.071 (0.025)	(−0.249, 0.391)	0.216	100.0	0.000	0.0	0.000	0.0	0.071
History of child abuse victimization	2	4	0.035 (0.041)	(−0.094, 0.164)	0.453	26.8	0.005^+^	73.2	0.000	0.0	0.035
* Sibling at school*	1	1	0.028 (0.020)	(−0.010, 0.067)	0.077	100.0	–	–	–	–	0.028
School domains (#4)											
Low quality of school/education	3	11	0.162 (0.089)	(−0.031, 0.354)	0.091^+^	9.4	0.018^***^	51.7	0.013	38.9	0.161
Negative school/class climate	3	15	0.148 (0.051)	(0.038, 0.259)	0.012^*^	37.2	0.000	0.1	0.007^+^	62.7	0.147^c^
* Large classes/schools*	1	1	0.145 (0.080)	(−0.011, 0.292)	0.035^*^	100.0	–	–	–	–	0.144^c^
Poor pupil-teacher relationship	4	10	0.128 (0.062)	(−0.013, 0.269)	0.071^+^	5.1	0.034^***^	94.9	0.000	0.0	0.127
Often changed schools	4	5	0.078 (0.063)	(−0.099, 0.254)	0.288	0.8	0.000	0.0	0.016^+^	99.2	0.078
Peer domains (#3)											
Involvement with truant/deviant peers	6	13	0.232 (0.015)	(0.200, 0.264)	<0.001^***^	42.7	0.002^+^	57.3	0.000	0.0	0.228^b^
Poor social competence	5	16	0.171 (0.085)	(−0.010. 0.351)	0.062^+^	5.1	0.009^*^	21.6	0.030^*^	73.3	0.169
Having many friends/being popular	2	10	0.096 (0.040)	(0.006, 0.186)	0.039^*^	2.3	0.015^***^	97.7	0.000	0.0	0.096^c^
* Multicultural peer group*	1	1	0.089 (0.020)	(0.050, 0.126)	<0.001^***^	100.0	–	–	–	–	0.088^c^

# *studies* number of studies, # *ES* number of effect sizes, *SE* standard error, *CI* confidence interval, *Sig* significance, *Meanz* mean effect size (Fisher’s *z*), % *Var* percentage of variance explained, *Level* 2 *variance* variance between effect sizes from the same study, *Level* 3 *variance* variance between studies, Mean *r* the correlation coefficient corresponding to the mean effect size *z*

Risk factors that could not be classified into one of the 42 created risk domains for dropout are presented in italics

^+^*p*  < 0.10; *p < 0.05; **p < 0.01; ***p < 0.001

^a^Significant large effect (according to the guidelines of Rice and Harris 2005)

^b^Significant medium effect (according to the guidelines of Rice and Harris 2005)

^c^Significant small effect (according to the guidelines of Rice and Harris 2005)

The estimated overall effect did not significantly deviate from zero for 19 risk domains. This implies that these domains cannot be regarded as risk domains for dropout. Three of these 19 risk domains showed a trend significant effect. Table 3 also shows the overall effects of 6 single risk factors (presented in *italics*). The factors “poor general well-being”, “adverse childhood experiences”, “age of mother (being younger)”, “large classes/schools” and “multicultural peer group” showed a significant medium to small overall effect size. The effect of the factor “sibling at school” was not significant, and could therefore not be identified as a risk factor for school dropout.

### Assessment of Bias

Table 4 presents the results of the three analyses that were conducted to assess bias in the estimated mean effect of each of the 43 risk domains for school absenteeism. There was no indication of bias in 13 estimated risk domain effects (i.e., 0 out of 3 methods indicated bias), some indication of bias in 22 risk domain effects (i.e., 1 out of 3 methods indicated bias), and moderate to strong indications of bias in 9 risk domain effects (i.e., 2 or 3 out of 3 methods indicated bias). These results show indications of bias in most of the estimated risk domains. For school dropout, no indication of bias was found in 14 estimated risk domain effects, some indication of bias in 20 risk domain effects, and moderate to strong indications of bias in 8 risk domain effects (see Table 5). Again, an indication of bias was found in most risk domains. For brevity, the funnel plots that were produced in the trim-and-fill analyses are not presented here, but are available upon request from the first author.

**Table 4:** Results of three methods for the assessment of bias in the estimated mean effects of the risk domains for school absenteeism

Domain of risk factors	*r*	Trim-and-fill analysis	Three-level Funnel Plot test	Three-level Egger”s regression test	Number of methods indicating bias (out of 3)
Child domains (#24)					
Age (being older)	0.126	–	β_1_ = −0.000, *p* = 0.699	β_1_ = 0.362, *p* = 0.675	0
Alcohol abuse	0.311	Overestimation (6 ES missing)	β_1_ = −0.000, *p* = 0.288	β_1_ = −5.162, *p* < 0.001^***^	2
Anti-social behavior/cognitions	0.428	Underestimation (23 ES missing)	β_1_ = −0.000, *p* = 0.080^+^	β_1_ = 1.539, *p* = 0.423	1
Anxiety	0.115	Overestimation (6 ES missing)	β_1_ = 0.000, *p* = 0.469	β_1_ = 0.443, *p* = 0.630	1
Being a sexual minority	0.273	Overestimation (1 ES missing)	β_1_ = −0.000, *p* = 0.102	β_1_ = 1.928, *p* = 0.177	1
Delinquent behavior	0.252	Overestimation (4 ES missing)	β_1_ = −0.000, *p* = 0.473	β_1_ = 4.497, *p* = 0.158	1
Depression	0.237	Overestimation (1 ES missing)	β_1_ = 0.000, *p* = 0.632	β_1_ = 0.359, *p* = 0.659	1
Drug abuse	0.327	Overestimation (6 ES missing)	β_1_ = −0.000, *p* = 0.770	β_1_ = 2.557, *p* = 0.291	1
Ethnicity (being non-white)	0.024	Overestimation (3 ES missing)	β_1_ = −0.000, *p* = 0.887	β_1_ = −0.544, *p* = 0.354	1
Having a job	0.071	–	β_1_ = −0.000, *p* < 0.001^***^	β_1_ = −34.826, *p* = 0.734	1
Having a negative school attitude	0.503	Underestimation (22 ES missing)	β_1_ = −0.000, *p* = 0.041^*^	β_1_ = −1.637, *p* = 0.669	2
High impact/negative life events	0.112	–	β_1_ = −0.000, *p* = 0.951	β_1_ = −0.571, *p* = 0.719	0
High sexual involvement	0.229	Overestimation (1 ES missing)	β_1_ = 0.000, *p* = 0.778	β_1_ = −12.814, *p* = 0.778	1
Low academic achievement	0.232	–	β_1_ = 0.000, *p* = 0.599	β_1_ = 1.471, *p* = 0.036^*^	1
Low academic self-concept	0.395	–	β_1_ = −0.000, *p* = 0.239	β_1_ = 2.604, *p* = 0.570	0
Low IQ/learning difficulties	0.099	Overestimation (1 ES missing)	β_1_ = −0.000, *p* = 0.895	β_1_ = 0.472, *p* = 0.659	1
Negative or no leisure activities	0.081	–	β_1_ = −0.000, *p* = 0.366	β_1_ = 2.769, *p* = 0.548	0
Not being religious	0.141	–	β_1_ = 0.000, *p* = 0.862	β_1_ = −0.417, *p* = 0.896	0
Other internalizing problems	0.307	Underestimation (11 ES missing)	β_1_ = 0.000, *p* = 0.032^*^	β_1_ = −1.286, *p* = 0.360	2
Poor physical health	0.178	–	β_1_ = −0.000, *p* = 0.781	β_1_ = 1.296, *p* = 0.110	0
Psychiatric symptoms/disorders	0.303	Overestimation (3 ES missing)	β_1_ = −0.000, *p* < 0.001^***^	β_1_ = 1.111, *p* = 0.184	2
Risky coping/personality profile	0.158	Overestimation (5 ES missing)	β_1_ = 0.000, *p* = 0.968	β_1_ = 0.094, *p* = 0.940	1
Showing risky behavior	0.226	–	β_1_ = −0.000, *p* < 0.001^***^	β_1_ = 11.517, *p* = 0.001^**^	2
Smoking	0.336	Overestimation (2 ES missing)	β_1_ = 0.000, *p* = 0.311	β_1_ = −20.214, *p* = 0.320	1
Family domains (#11)					
Family structure (no nuclear family)	0.187	Underestimation (7 ES missing)	β_1_ = −0.000, *p* = 0.474	β_1_ = 1.368, *p* = 0.466	1
History of child abuse victimization	0.257	–	β_1_ = −0.000, *p* = 0.863	β_1_ = 1.492, *p* = 0.342	0
Ineffective family systems	0.153	–	β_1_ = −0.000, *p* = 0.270	β_1_ = 0.534, *p* = 0.673	0
Large family size	0.194	Overestimation (1 ES missing)	β_1_ = −0.000, *p* = 0.094^+^	β_1_ = 2.304, *p* = 0.094^+^	1
Low family SES	0.134	Overestimation (5 ES missing)	β_1_ = −0.000, *p* = 0.119	β_1_ = 1.305, *p* = 0.019^*^	2
Low parental control	0.123	–	β_1_ = 0.000, *p* = 0.371	β_1_ = −0.980, *p* = 0.105	0
Low parental education	0.155	Underestimation (6 ES missing)	β_1_ = −0.000, *p* = 0.063^+^	β_1_ = 0.676, *p* = 0.569	1
Low parental school involvement	0.272	Underestimation (5 ES missing)	β_1_ = −0.000, *p* = 0.008^**^	β_1_ = 2.189, *p* = 0.156	2
Low parental support/acceptance	0.182	Underestimation (1 ES missing)	β_1_ = 0.000, *p* = 0.571	β_1_ = −0.480, *p* = 0.502	1
Parental mental/physical problems	0.186	–	NA	NA	0
Sibling at school	0.065	Overestimation (1 ES missing)	NA	NA	1
School domains (#6)					
Distance to school (short)	0.476	–	NA	NA	0
Large classes/schools	0.044	Underestimation (2 ES missing)	β_1_ = 0.000, *p* = 0.903	β_1_ = −0.739, *p* = 0.432	1
Low quality of school/education	0.229	Underestimation (2 ES missing)	β_1_ = −0.000, *p* = 0.111	β_1_ = 2.955, *p* = 0.024^*^	2
Negative school/class climate	0.183	Underestimation (5 ES missing)	β_1_ = −0.000, *p* = 0.435	β_1_ = −0.263, *p* = 0.827	1
Poor pupil-teacher relationship	0.286	Underestimation (2 ES missing)	β_1_ = −0.000, *p* = 0.207	β_1_ = 0.319, *p* = 0.875	1
Public school (vs. private)	0.098	Overestimation (1 ES missing)	β_1_ = −0.000, *p* = 0.002^**^	β_1_ = 6.808, *p* = 0.002^**^	3
Peer domains (#3)	0.056				
Being bullied	0.011	Overestimation (2 ES missing)	β_1_ = 0.000, *p* = 0.891	β_1_ = −0.883, *p* = 0.709	1
Having many friends	0.201	–	β_1_ = −0.001, *p* = .208	β_1_ = 4.279, *p* = 0.234	0
Poor social competence	0.106	–	β_1_ = −0.001, *p* = 0.789	β_1_ = 1.352, *p* = 0.491	0

*r* mean effect size (Pearson”s correlation; see also Table 2), *Underestimation* effect sizes were imputed to the right of the mean effect, implying that above average effect sizes were underrepresented and that the mean effect may be an underestimation of the true effect, *Overestimation* effect sizes were imputed to the left of the mean effect, implying that below average effect sizes were underrepresented and that the mean effect may be an overestimation of the true effect, NA not available, as only two or three effect sizes were classified in the risk domain

Dashes indicate that trimming and filling of effect sizes were not necessary according to the trim-and-fill algorithm

^+^*p<* 0.10; ^*^p < 0.05; ^**^p < 0.01; ^***^p < 0.001

**Table 5:** Results of three methods for the assessment of bias in the estimated mean effects of the risk domains for school dropout

Domain of risk factors	*r*	Trim-and-fill analysis	Three-level Funnel Plot test	Three-level Egger”s regression test	Number of methods indicating bias (out of 3)
Child domains (#23)					
Age (Being older)	0.195	Underestimation (2 ES missing)	β_1_ = 0.000, *p* = 0.308	β_1_ = −2.688, *p* = 0.043^*^	2
Alcohol abuse	0.101	Underestimation (1 ES missing)	β_1_ = −0.003, *p* < 0.001^***^	β_1_ = 32.531, *p* < 0.001^***^	3
Anti-social behavior/cognition	0.236	–	β_1_ = −0.000, *p* = 0.065^+^	β_1_ = 1.293, *p* = 0.330	0
Anxiety	0.009	–	β_1_ = −0.000, *p* = 0.449	β_1_ = 4.580, *p* = 0.449	0
Delinquent behavior	0.223	Overestimation (3 ES missing)	β_1_ = −0.000, *p* = 0.568	β_1_ = 0.791, *p* = 0.592	1
Depression	0.069	–	β_1_ = 0.000, *p* = 0.772	β_1_ = −0.977, *p* = 0.731	0
Drug abuse	0.247	Overestimation (2 ES missing)	β_1_ = −0.000, *p* = 0.283	β_1_ = 2.162, *p* = 0.098^+^	1
Ethnicity (being non-white)	0.062	Overestimation (11 ES missing)	β_1_ = −0.000, *p* = 0.668	β_1_ = −0.163, *p* = 0.847	1
Having a job	0.088	Underestimation (1 ES missing)	NA	NA	1
Having a negative school attitude	0.210	Underestimation (9 ES missing)	β_1_ = 0.000, *p* = 0.118	β_1_ = −1.260, *p* = 0.411	1
High sexual involvement	0.170	–	β_1_ = 0.000, *p* = 0.662	β_1_ = −6.660, *p* = 0.465	0
History of grade retention	0.348	Underestimation (1 ES missing)	β_1_ = 0.000, *p* = 0.109	β_1_ = 1.169, *p* = 0.617	1
Low academic achievement	0.316	Underestimation (20 ES missing)	β_1_ = −0.000, *p* = 0.949	β_1_ = 1.247, *p* = 0.202	1
Low academic self-concept	0.217	Underestimation (2 ES missing)	β_1_ = 0.000, *p* = 0.004^**^	β_1_ = −6.891, *p* = 0.060^+^	2
Low IQ/learning difficulties	0.326	–	β_1_ = −0.000, *p* = 0.509	β_1_ = 3.641, *p* = 0.011^*^	1
Negative or no leisure activities	0.084	–	β_1_ = 0.000, *p* = 0.031^*^	β_1_ = −3.170, *p* = 0.422	1
Not being religious	0.113	Underestimation (1 ES missing)	NA	NA	1
Other internalizing problems	0.140	Underestimation (1 ES missing)	β_1_ = −0.000, *p* = 0.720	β_1_ = 0.338, *p* = 0.937	1
Poor physical health	0.157	Underestimation (1 ES missing)	β_1_ = −0.000, *p* = 0.726	β_1_ = 3.336, *p* = 0.399	1
Psychiatric symptoms/disorders	0.269	–	β_1_ = 0.000, *p* = 0.171	β_1_ = −2.673, *p* = 0.171	0
Risky coping/personality profile	0.057	–	β_1_ = −0.000, *p* = 0.158	β_1_ = 0.216, *p* = 0.887	0
Showing risky behavior	0.109	–	NA	NA	0
Smoking	0.125	–	β_1_ = 0.000, *p* = 0.002^**^	β_1_ = −20.443, *p* = 0.002^**^	2
Family domains (#12)					
Family structure (no nuclear family)	0.178	–	β_1_ = 0.000, *p* = 0.760	β_1_ = −1.078, *p* = 0.192	0
History of child abuse victimization	0.035	Underestimation (1 ES missing)	β_1_ = −0.000, *p* = 0.547	β_1_ = 4.289, *p* = 0.547	1
Ineffective family systems	0.180	–	β_1_ = 0.001, *p* = 0.161	β_1_ = −40.624, *p* = 0.117	0
Large family size	0.194	–	β_1_ = −0.000, *p* = 0.295	β_1_ = 3.813, *p* = 0.446	0
Low family SES	0.222	–	β_1_ = 0.000, *p* = 0.305	β_1_ = 2.197, *p* = 0.044^*^	1
Low parental control	0.134	–	β_1_ = −0.000, *p* = 0.123	β_1_ = 3.240, *p* = 0.149	0
Low parental education	0.200	–	β_1_ = 0.000, *p* = 0.320	β_1_ = −3.383, *p* = 0.136	0
Low parental school involvement	0.149	Underestimation (2 ES missing)	β_1_ = −0.000, *p* = 0.262	β_1_ = −1.702, *p* = 0.284	1
Low parental support/acceptance	0.176	Underestimation (2 ES missing)	β_1_ = 0.000, *p* = 0.032^*^	β_1_ = −2.108, *p* = 0.216	2
Parental alcohol use	0.071	Underestimation (1 ES missing)	NA	NA	1
Poor parent-child relationship	0.164	–	β_1_ = −0.000, *p* = 0.390	β_1_ = 3.133, *p* = 0.097^+^	0
Sibling dropped out	0.121	Underestimation (1 ES missing)	NA	NA	1
School domains (#4)					
Low quality of school/education	0.161	–	β_1_ = −0.000, *p* = 0.568	β_1_ = 2.238, *p* = 0.464	0
Negative school/class climate	0.147	–	β_1_ = 0.000, *p* < 0.001^***^	β_1_ = −1.834, *p* < 0.001^***^	2
Often changed schools	0.127	–	β_1_ = 0.000, *p* = 0.002^**^	β_1_ = −5.973, *p* = 0.022^*^	2
Poor pupil-teacher relationship	0.286	Underestimation (1 ES missing)	β_1_ = 0.000, *p* = 0.612	β_1_ = −0.429, *p* = 0.840	1
Peer domains (#3)	0.056				
Having many friends	0.096	Underestimation (2 ES missing)	β_1_ = −0.000, *p* = 0.781	β_1_ = 1.345, *p* = 0.781	1
Involvement with truant/deviant peers	0.228	Underestimation (3 ES missing)	β_1_ = 0.000, *p* = 0.247	β_1_ = −2.817, *p* = 0.247	1
Poor social competence	0.169	Overestimation (3 ES missing)	β_1_ = −0.000, *p* = 0.225	β_1_ = 4.967, *p* = 0.029^*^	2

*r* mean effect size (Pearson”s correlation; see also Table 3), *Underestimation* effect sizes were imputed to the right of the mean effect, implying that above average effect sizes were underrepresented and that the mean effect may be an underestimation of the true effect, *Overestimation* effect sizes were imputed to the left of the mean effect, implying that below average effect sizes were underrepresented and that the mean effect may be an overestimation of the true effect, *NA* not available, because only two effect sizes were classified in the corresponding risk domain, or because all effect sizes classified in the risk domain originated from one study

Dashes indicate that trimming and filling of effect sizes were not necessary according to the trim-and-fill algorithm

^+^*p<* 0.10; ^*^p < 0.05; ^**^p < 0.01; ^***^p < 0.001

### The Moderating Effect of Gender

Table 2 shows the results of the likelihood-ratio tests that were performed to examine heterogeneity in effect sizes in the school absenteeism risk domains. In 37 risk domains, there was significant level-2 and/or level-3 variance. In the risk domains “psychiatric symptoms/disorders”, “low IQ/learning difficulties”, “large family size”, “sibling at school”, “distance to school (short)”, and “having many of friends”, there was no indication for heterogeneity in effect sizes. Therefore, no moderator analyses were performed in these domains. Further, and as mentioned in the Method section, the percentage of boys was only tested as a moderator when this variable was based on at least five studies. In the end, moderator analyses were performed for 20 risk domains for school absenteeism, and the results are presented in Table 6. A significant moderating effect was only found in the risk domain “drug abuse”, showing that the effect of this domain decreased as the percentage of boys in samples increased.

**Table 6:** Results of testing gender as a potential moderator in various risk domains for school absenteeism

Moderator variable: Percentage of boys in the sample	# Studies	# ES	Intercept (95% CI)/ Mean *z* (95% CI)	β_1_ (95% CI)	*F* (df1, df2)^a^	*p* ^b^	Level 2 variance	Level 3 variance
Child domains								
Age (Being older)	11	15	0.127 (0.058, 0.196)^**^	−0.588 (−2.143, 0.967)	0.667 (1, 13)	0.429	0.002^***^	0.008
Alcohol abuse	7	35	0.277 (0.097, 0.458)^**^	0.463 (−4.403, 5.329)	0.038 (1, 33)	0.848	0.020^***^	0.032
Anti-social behavior/cognitions	14	38	0.249 (0.183, 0.316)^***^	0.500 (−0.654, 1.654)	0.771 (1, 36)	0.386	0.019^***^	0.006
Anxiety	9	25	0.106 (0.010, 0.202)^*^	−0.674 (−1.905, 0.557)	1.282 (1, 23)	0.269	0.007^***^	0.012^**^
Delinquent behavior	5	16	0.101 (−0.223, 0.425)	−0.747 (−5.342, 3.848)	0.221 (1, 14)	0.732	0.015^***^	0.103^***^
Depression	8	13	0.239 (0.168, 0.311)^***^	0.278 (−0.861, 1.417)	0.288 (1, 11)	0.602	0.009^***^	0.000
Drug abuse	7	24	0.401 (0.291, 0.512)^***^	−2.229 (−3.907, −0.551)^*^	7.587 (1, 22)	0.012^*^	0.010^***^	0.012^**^
Ethnicity (Being non-white)	12	29	0.038 (−0.027, 0.103)	0.038 (−0.086, 0.162)	0.397 (1.27)	0.534	0.007^***^	0.007
Having a negative school attitude	8	50	0.304 (0.180, 0.427)^***^	0.987 (−0.949, 2.923)	1.050 (1, 48)	0.311	0.002^***^	0.020^***^
Low academic achievement	9	19	0.225 (0.104, 0.347)^**^	−0.114 (−0.851, 0.622)	0.107 (1, 17)	0.748	0.021^***^	0.014
Low IQ/learning difficulties	5	6	0.107 (0.003, 0.211)^*^	−0.267 (−1.482, 0.947)	0.374 (1, 4)	0.574	0.006	0.000
Other internalizing problems	8	18	0.172 (0.071, 0.274)^*^	−0.709 (−2.334, 0.915)	0.856 (1, 16)	0.369	0.011^***^	0.009
Poor physical health	10	57	0.179 (0.087, 0.272)^***^	0.056 (−1.292, 1.403)	0.007 (1, 55)	0.934	0.010^***^	0.015^***^
Risky coping/personality profile	5	30	0.158 (0.111, 0.204)^***^	0.085 (−1.239, 1.408)	0.017 (1, 28)	0.897	0.011^***^	0.000
Family domains								
Family structure	10	23	0.179 (0.081, 0.278)^**^	0.959 (−1.474, 3.393)	0.672 (1, 21)	0.421	0.050^***^	0.000
Low family SES	15	35	0.088 (−0.007, 0.182)^+^	0.398 (−1.170, 1.966)	0.267 (1, 33)	0.609	0.008^***^	0.024^***^
Low parental control	5	12	0.110 (0.010, 0.210)^*^	0.137 (−0.985, 1.259)	0.074 (1, 10)	0.791	0.005^***^	0.006
Low parental education	6	17	0.156 (0.063, 0.249)^**^	−0.457 (−2.055, 1.141)	0.372 (1, 15)	0.551	0.012^***^	0.004
School domains								
Negative school/class climate	6	22	0.184 (0.108, 0.260)^***^	−2.642 (−7.892, 2.608)	1.102 (1, 20)	0.306	0.019^***^	0.001
Peer domains								
Poor social competence	5	7	0.171 (−0.036, 0.378)^+^	−1.523 (−4.805, 1.759)	1.423 (1, 5)	0.286	0.038^***^	0.000

# Studies number of studies, # *ES* number of effect sizes, *Meanz* mean effect size (z), *CI* confidence interval, β_1_ estimated regression coefficient, *Level* 2 *variance* residual variance between effect sizes from the same study, *Level* 3 *variance* residual variance between studies

^+^*p*  < 0.10; *p < 0.05; **p < 0.01; ***p < 0.001

^a^Omnibus test of all regression coefficients in the model

^b^*p*-Value of the omnibus test

Table 3 shows the results of the likelihood-ratio tests for the school dropout risk domains. Significant level-2 and/or level-3 variance was found in 32 risk domains. There was no indication for heterogeneity in effect sizes in the risk domains “delinquent behavior”, “not being religious”, “having a job”, “anxiety”, “large family size”, “parental alcohol use”, “history of child abuse victimization”, “negative school/class climate”, “often changed schools”, and “involvement with truant/deviant peers”. Also taking into account the lower bound that was set to five studies (see Method section), the percentage of boys was tested as a moderator in 15 risk domains for school dropout. The results are presented in Table 7, and reveal that only the overall effect of “having a negative school attitude” was moderated by gender. This finding implied that the effect of this risk domain for dropout decreased as the percentage of boys in samples increased.

**Table 7:** Results of testing gender as a potential moderator in various risk domains for school dropout

Moderator variable: Percentage of boys in the sample	# Studies	# ES	Intercept (95% CI)/ Mean *z* (95% CI)	β_1_ (95% CI)	*F* (df1, df2)^a^	*p* ^b^	Level 2 variance	Level 3 variance
Child domains								
Anti-social behavior/cognitions	13	33	0.233 (0.153, 0.313)^***^	0.148 (−0.080, 0.376)	1.745 (1, 31)	0.196	0.007^***^	0.015^**^
Drug abuse	5	9	0.272 (0.166, 0.378)^***^	0.616 (−0.064, 1.295)^+^	4.593 (1, 7)	0.069^+^	0.012^***^	0.000
Ethnicity (Being non-white)	11	50	0.043 (−0.003, 0.089)^+^	0.575 (−2.313, 3.462)	0.160 (1, 48)	0.691	0.019^***^	0.001
Having a negative school attitude	9	44	0.208 (0.161, 0.255)^***^	−1.521 (−3.023, −0.019)^*^	4.175 (1, 42)	0.047^*^	0.023^***^	0.000
History of grade retention	10	18	0.362 (0.255, 0.470)^***^	−0.031 (−0.493, 0.431)	0.020 (1, 16)	0.890	0.023^***^	0.010
Low academic achievement	19	87	0.341 (0.270, 0.412)^***^	0.017 (−0.111, 0.144)	0.068 (1. 85)	0.795	0.024^***^	0.013^*^
Low IQ/learning difficulties	7	11	0.362 (0.195, 0.529)^***^	0.390 (−0.243, 1.024)	1.942 (1, 9)	0.197	0.054^***^	0.000
Risky coping/personality profile	5	20	0.058 (−0.015, 0.131)	−0.067 (−0.427, 0.292)	0.156 (1, 18)	0.698	0.022^***^	0.000
Family domains								
Family structure	7	17	0.173 (0.120, 0.226)^***^	−0.061 (−0.222, 0.099)	0.660 (1, 15)	0.429	0.008^***^	0.000
Low family SES	14	30	0.233 (0.131, 0.335)^***^	0.095 (−0.206, 0.396)	0.418 (1, 28)	0.523	0.011^***^	0.027^***^
Low parental control	8	21	0.124 (0.004, 0.244)^*^	0.168 (−0.118, 0.454)	1.516 (1, 19)	0.233	0.004^***^	0.022^***^
Low parental education	10	20	0.203 (0.120, 0.287)^***^	−0.027 (−0.166, 0.113)	0.162 (1, 18)	0.692	0.003^***^	0.013^**^
Low parental school involvement	6	24	0.151 (0.058, 0.245)^**^	0.141 (−0.086, 0.368)	1.655 (1, 22)	0.212	0.010^***^	0.009^**^
Low parental support/acceptance	8	16	0.177 (0.064, 0.289)^**^	0.029 (−0.262, 0.320)	0.045 (1, 14)	0.834	0.014^***^	0.013
Peer domains								
Involvement with truant/deviant peers	5	12	0.226 (0.195, 0.258)^***^	0.181 (−0.037, 0.399)	3.433 (1, 10)	0.094^+^	0.001^+^	0.000

# Studies number of studies, # *ES* number of effect sizes, *Meanz* mean effect size (z), *CI* confidence interval, β_1_ estimated regression coefficient, *Level* 2 *variance* residual variance between effect sizes from the same study, *Level* 3 *variance* residual variance between studies

^+^*p* < 0 .10; *p < 0.05; **p < 0 .01; ***p < 0.001

^a^Omnibus test of all regression coefficients in the model

^b^*p*-Value of the omnibus test

## Discussion

A great amount of literature has reported on potential risk factors for school absenteeism and/or school dropout, but a systematic review summarizing effects of risk factors for school absenteeism and risk factors for dropout was not yet available. Therefore, the aim of the present study was to estimate a mean effect of various risk domains (i.e., groups of more or less similar risk factors) for school absenteeism and various risk domains for school dropout. Both these constructs were examined in this meta-analytic review, as youths with excessive absenteeism are at high risk for permanent dropout from school (i.e., Kearney 2008a) and therefore, the constructs may share various risk factors. However, it is also relevant to examine whether and how risk factors for school absenteeism differ from risk factors for school dropout. The second aim of this study was to examine whether the percentage of boys in samples moderates the overall strength of individual risk domains for school absenteeism or dropout.

### Overall Effect of Risk Domains

The results revealed that multiple child-, family-, school- and peer-related risk factors contribute to the risk for both school absenteeism and school dropout. This is in line with the interdisciplinary model of school absenteeism formulated by Kearney (2008a), in which several types of school absenteeism are influenced by various factors, including child, parent, family, peer, school, and community variables.

For school absenteeism, a significant overall effect was found for 28 out of 44 examined risk domains, ranging from *r=* 0.099 for having a low IQ or experiencing learning difficulties to *r=* 0.553 for having a negative school attitude. Large effects were found for 11 risk domains, including risks related to having a negative attitude towards school, substance abuse, externalizing and internalizing problem behavior of the child, and a low involvement of parents in school. For ease of interpretation, a number of “risk themes” were formulated that capture all significant risk domains (see also Assink et al. 2019 who applied this procedure in their review on risk factors for victimization of child sexual abuse). Given the current results, seven themes could be identified. First, the results indicate that moderate to large effects were found for multiple risk domains related to *physical and mental problems* of the child, which were: showing problematic internalizing behavior (other than being depressed and having anxieties; *r=* 0.307), having psychiatric symptoms or disorders (*r* = 0.303), being depressed (*r* = 0.237), having a poor physical health (*r* = 0.178), and suffering from anxieties (*r* = 0.115). Related to this theme, it was secondly found that risks referring to *substance abuse* had large effects, including smoking (*r=* 0.336), drug abuse (*r* = 0.327), and alcohol abuse (*r=* 0.311). Third, several *antisocial or risky behaviors* of the child were identified as risk factors, including showing anti-social behavior or having anti-social cognitions (*r=* 0.428), a high sexual involvement (*r* = 0.229), showing risky behavior (such as risky behavior in traffic; *r* = 0.226), and showing ineffective coping or having a risky personality profile (*r* = 0.158). Fourth, it was found that multiple risk domains relate to different sorts of *problems at or with school*, including having a negative school attitude (*r* = 0.503), a poor teacher-pupil relationship (*r* = 0.286), low levels of academic achievement (*r* = 0.232), a history of grade retention (*r* = 0.100), and a low IQ or learning difficulties (*r=* 0.099). Related to this theme are different *characteristics of the school* that pose a risk for absenteeism, including a low quality of the school or education (*r=* 0.229) and a negative school or class climate (*r* = 0.183). Sixth, *parenting problems and difficulties* are also important risk factors for school absenteeism, as significant effects were found of parents showing low levels of school involvement (*r* = 0.272), a low parental attachment (*r* = 0.220), parental mental or physical problems (*r=* 0.186), low levels of parental support or acceptance (*r=* 0.182), and low levels of parental control (*r* = 0.123). Finally, *family (structure) problems* could also be designated as significant risks, including a history of child abuse victimization in the family (*r* = 0.257), a non-nuclear family structure (*r* = 0.187), a low level of parental education (*r=* 0.155), an ineffective family system (*r* = 0.154), and a low family SES (*r* = 0.134).

For school dropout, a significant overall effect in a positive direction was found for 23 out of 42 risk domains. Large effects were found for the risk factors having a history of grade retention (*r=* 0.348), having a low IQ or experiencing learning difficulties (*r=* 0.326) and showing low levels of academic achievement (*r=* 0.316). For the dropout risk domains and the significant individual risk factors seven risk themes could be identified, with six themes being similar to those formulated for school absenteeism. First, *problems at or with school* were important risks for dropout. Medium to large effects were found for the risk domains having a history of grade retention (*r* = 0.348), having a low IQ or learning difficulties (*r* = 0.326), low levels of academic achievement (*r* = 0.316), and having a negative school attitude (*r=* 0.210). The second risk theme consist of *physical and mental problems* of the child, such as: having psychiatric problems or disorders (*r* = 0.269), abusing drugs (*r=* 0.247), poor general well-being (*r* = 0.210), having adverse childhood experiences (*r=* 0.185), poor physical health (*r* = 0.157), and internalizing behavior problems (other than being depressed or having anxieties; *r* = 0.140). Third, several *anti-social behaviors* were identified as risk factors for school dropout, including showing anti-social behavior or having anti-social cognitions (*r* = 0.236), engaging in delinquent behavior (*r* = 0.223), showing risky behaviors (*r=* 0.109), and being involved with truant or deviant peers (*r* = 0.228). Fourth, *parenting problems and difficulties* were found to be important risk factors for school dropout, including low levels of parental support or acceptance (*r* = 0.176), low levels of parental involvement in school (*r* = 0.149), and low levels of parental control (*r=* 0.134) Fifth, other *family (structure) problems* could be designated as significant risks, as significant effects were found for a low family SES (*r* = 0.222), a low educational level of parents (*r=* 0.200), large families (*r=* 0.194), and a non-nuclear family structure (*r=* 0.178). Sixth, school dropout was related to *characteristics of the school* such as a negative climate in school or class (*r* = 0.147) and large schools or classes (*r=* 0.145). Finally, the results showed that *peer group characteristics or social status within a peer group* had small significant effects on school dropout, including having many friends or being popular (*r* = 0.096) and being involved in a multicultural peer group (*r* = 0.088). This final risk theme is unique for school dropout. Naturally, the involvement with truant or deviant peers, which is was previously mentioned as part of the risk theme related to the anti-social behaviors of the child, can also be regarded as part of this final risk theme.

The abovementioned risk themes for school absenteeism and dropout are largely similar in nature, suggesting that both school absenteeism and dropout are related to similar risk factors. This was in line with what could be expected, because young people showing excessive absenteeism are at high risk for permanent school dropout. In his interdisciplinary model, Kearney (2008a) suggests that several factors influence problematic school absenteeism, which could deteriorate over time from an acute, to a chronic, to a permanent state (dropout) of absenteeism. Moreover, since school drop-out is a more serious form of school absenteeism, it is possible that dropping out of school mainly results from an accumulation of multiple (different) risk factors, whereas the presence of a single (strong) risk factor may already lead to school absenteeism. This is also in line with the findings of Suh et al. (2007) indicating that as risk factors accumulate, students are more likely to drop out of school.

### Moderating Effect of Gender

The variable percentage of boys in samples of primary studies was examined as a potential moderator of the overall strength of risk domains in which heterogeneity in effect sizes was identified. For school absenteeism, the effect of abusing drugs increased as the percentage of boys in samples decreased. This means that abusing drugs is a stronger risk factor for school absenteeism in girls than in boys. Previous research indicates that drug abuse rates are higher in men than in women (e.g., Becker and Hu 2008; Center for Behavioral Health Statistics and Quality 2017). Therefore, it is possible that drug abuse in boys is perceived as “more normal” or less deviant than in girls. This may imply that drug abuse contributes more to the risk of school absenteeism in girls than in boys.

For school dropout, it was found that only the effect of having a negative school attitude was moderated by the percentage of boys in primary study samples. The effect of this risk domain decreased as the percentage of boys increased, which means that having a negative school attitude is a stronger predictor of school dropout in girls than in boys. Prior research has revealed that boys have a more negative attitude towards school than girls (e.g., Harvey 1985; Logan and Johnston 2009). This negative attitude may stem from the fact that most school environments are centered around group and team work, whereas school environments in which autonomy is fostered (e.g., authority, aggression, and technical competence; Daniels et al. 2001) would better fit a masculine orientation to learning. As girls are generally less negative about school, it may be that girls with a negative school attitude may have to deal with other risk factors that are related to this negative attitude. Therefore, a negative school attitude might contribute more to the risk of school dropout in girls than in boys. It must be noted that most risk domains were not moderated by gender, indicating that the effect of most risk domains for school absenteeism and dropout seem similar for boys and girls.

### Limitations

Several limitations of the present study should be mentioned. First, despite an extensive search procedure, it cannot be assured that the current sample of included studies is representative of all studies on (putative) risk factors for school absenteeism and dropout. A large amount of literature is available on the effect of risk factors for school absenteeism and dropout, and therefore it is possible that primary studies were missed. However, given the current extensive data set (a total of 69 studies and 1384 effect sizes), it may be assumed that the included studies were sufficiently representative of all primary studies available on risk factors for school absenteeism and dropout. Furthermore, the study inclusion was restricted to published studies and dissertations, there was a risk for overestimating effects of risk domains due to publication bias. The three tests for bias assessment indicated that bias may have been present in multiple estimated effects of risk domains. However, trim-and-fill analyses showed that an underestimation rather than an overestimation of risk domain effects was a problem (see Tables 4 and 5). Therefore, bias in the analyzes data may not be due to specifically publication bias.

Second, the present study does not permit conclusions about causality between the presence of a risk factor and school absenteeism or dropout, because of the non-experimental nature of the included studies. In addition, in extracting effects of (putative) risk factors from primary studies, there was a focus on antecedents of school absenteeism and dropout (see also the inclusion criteria mentioned in the Method section), but as many included studies were retrospective in nature, it cannot be assured that all factors classified into the risk domains were true antecedents rather than outcomes. Further, it has been acknowledged that risk factors for school absenteeism and dropout are not present in isolation, but coexist and interact with other risk factors (e.g., Berends and Diest 2014; Ingul et al. 2012; Kearney 2008a, 2008b). However, in the main focus of the present study was the mean effect of individual risk domains, and each risk factor was therefore classified into one of mutually exclusive risk domains. This allowed conducting a separate meta-analysis for each risk domain in order to estimate the mean effect of groups of (more or less) similar risk factors for school absenteeism and dropout. However, this did not allow us to examine what combinations of risk domains (or risk factors) may especially be predictive for school absenteeism and dropout. This may be a focus in future youth and adolescence research.

Finally, in the analytic strategy used this study, it was decided to only examine the variable percentage of boys in samples of primary studies as a potential moderator of risk domain effects. This decision was made as performing a large number of moderator analyses is not only impractical, but also statistically unwise, as insufficient data and capitalization on chance pose important problems. Furthermore, it was decided to only perform moderator analyses for variables that were based on at least five studies. Most coded variables did not meet this criterion, as some risk domains consisted of a small number of studies and effect sizes. As it was decided to only examine one potential moderator, the current study does not elaborate on the potential differences in overall effects of risk domains across different study designs or children with different background characteristics (e.g., age). Therefore, future youth and adolescence research should focus on examining effects of specific risk factors in different groups and under different circumstances.

### Implications of the Study

The current study has a number of important implications. First, the current findings contribute to the fundamental knowledge of the etiology of school absenteeism and dropout, which in turn contributes to a better understanding of the problematic development of adolescents. Based on earlier research, it was already known that school absenteeism and dropout are caused by multiple child, parent, family, peer, and school factors. This study adds knowledge about which factors are most important in the etiology of both school absenteeism and dropout. This is important knowledge, for example for school professionals, that can be used in detecting risks of school absenteeism and dropout at an early stage, and in providing adequate prevention.

Furthermore, proper risk and needs assessment is essential in answering which children are at risk for school absenteeism or dropout and may therefore benefit from an (preventive) intervention. Risk and needs assessment may also indicate what factors should be targeted in an intervention so that the risk for school absenteeism or dropout could be reduced. So far, only measures have been developed to assess child factors among youth with specific types of school absenteeism, such as school refusal behavior (Kearney 2002; Kearney and Silverman 1993) and truancy (Kim and Barthelemy 2010). It was found that various child-, family-, school-, and peer-related risks are related to school absenteeism and dropout. Therefore, the results of this review show that the risk for school absenteeism and dropout can best be assessed from a multifactorial perspective in future risk- and need assessment instruments. This is in line with the suggestion of a multiaxial assessment of risk factors by Kearney (2008a). Practitioners should focus on the assessment of factors related to the abovementioned risk themes, as it was found that these themes describe the risks that are predictive for school absenteeism and dropout. Furthermore, the risk domains with high overall effects on school absenteeism, including risks related to substance abuse and externalizing behavior, were most predictive and therefore deserve specific attention within risk- and need assessment instrument. Assessment instruments for school dropout should specifically focus on the child’s IQ, learning difficulties of the child, and a history of grade retention. As permanent dropout is often the consequence of excessive school absenteeism (Kearney 2008a), it can be argued to assess both school absenteeism and dropout in a single instrument, while taking into account the differences in impact between school absenteeism risk factors and dropout risk factors. Furthermore, the findings of this review can be used to improve the validity of risk and needs assessment tools, as these findings indicate which risk factors are most strongly related to school absenteeism and dropout and should therefore be assessed by these tools. Assessing more relevant risk factors increases the validity of risk and needs assessment instruments.

As for the broad and multifactorial perspective that is needed in risk and needs assessment, (preventive) interventions should also be based on the notion that school absenteeism and dropout results from the presence of multiple child-, family-, school-, and peer-related factors. This means that all these factors should be taken into account in order to effectively reduce or prevent school absenteeism and dropout. Further, previous review studies indicate an insufficient effect of currently available intervention and preventions programs (Maynard et al. 2013; Wilson and Tanner-Smith 2013). This indicates a need for more effective interventions, for which the current findings may serve as a foundation.

## Conclusion

School absenteeism and dropout are associated with many different life-course problems. To reduce the risk for these problems it is important to gain insight into risk factors for both school absenteeism and permanent school dropout. Until now, no quantitative overview of these risk factors and their effects was available. Therefore, this study was aimed at meta-analytically synthesizing the available evidence on risk factors for school absenteeism and dropout. The results of this study revealed that a substantial number of risks contribute to school absenteeism and dropout. For school absenteeism, significant and substantial effects were found for risks that refer to: physical and mental problems of the child (e.g., having psychiatric symptoms or disorders), substance abuse (e.g., drug abuse), antisocial or risky behavior (e.g., showing anti-social behavior or having anti-social cognitions), problems at or with school (e.g., having a negative school attitude), characteristics of the school (e.g., low quality of the school or education), parenting problems and difficulties (e.g., low parental school involvement), and family problems (e.g., an ineffective family system). As for school dropout, similar risks were identified next to risks related to peer group characteristics or social status in a peer group. The results imply that a multifactorial approach is needed in risk and needs assessment, and in interventions aimed at reducing or preventing school absenteeism and dropout. This review provides valuable insights for the development and improvement of both assessment and (preventive) intervention strategies.

## Appendix A

Examples of risk factors classified in each risk domain

**Table:**

Child domains
**Age (Being older; A** **+** **D)**
Age of child (older = more risk); Grade level of child (higher grade = more risk)
**Alcohol abuse (A** **+** **D)**
Child’s lifetime alcohol use; Child had ever used alcohol; Child is a heavy drinker; Child is often drunk; Child started drinking alcohol early in life; Child has problems because of alcohol use
**Anti-social behavior/cognitions (A** **+** **D)**
Child is aggressive; Child is anti-social (but not delinquent); Child has anti-social orientation; Child has attention problems; Child has behavioral problems; Child has attitudinal problems; Child shows disruptive behavior; Child is violent; Child has conduct problems; Child has disciplinary referrals at school; Child is a bully; Child is hyperactive; Child is irresponsible; Child is prone to mischief; Child shows a lot of anger or irritability Child shows rule breaking behavior; Child has low self-control
**Anxiety (A** **+** **D)**
Child shows generalized anxiety/anxiety symptoms/separation anxiety/simple phobia/social anxiety
**Being a sexual minority (A)**
Being bisexual, lesbian, gay or unsure about sexual identity
**Delinquent behavior (A** **+** **D)**
Child has committing school crime; Child shows vandalism; Child was arrested; Child carries a gun or weapon; Child has a criminal history; Child is delinquent; Child committed a violent offense; Child sells drugs; Child was in jail; Child steals; Child showed weapon violence; Child was in juvenile probation
**Depression (A** **+** **D)**
Child has as history of depression or is currently depressed
**Drug abuse (A** **+** **D)**
Child is using or used methamphetamine/marijuana/ecstasy/cocaine/steroid/illicit drugs/inhalant drugs/other narcotics
**Ethnicity (Being non-White; A** **+** **D)**
Child is Asian/African American/Native American/Hispanic/non-white/non-Western/multiracial/a minority/an immigrant; English is child’s second language (in studies from English-speaking countries); Dutch is child’s second language (in studies from the Netherlands)
**Having a job (A** **+** **D)**
Child is employed; Child is working for money; Child worked in past year
**Having a negative school attitude (A** **+** **D)**
Child dislikes school; Child had s academic disinterest; Child does little homework; Child does not understand the purpose of schooling; Child perceives school grades as unimportant; Child had a history of dropping out; Child doesn’t feel a part of the school community; Child is often late in class; Child show low levels of school engagement; Child shows low attachment to school; Child is not committed to school; Child has low educational goals; Child shows a low motivation; Child has a negative attitude towards school; Child is not sure of high school graduation
**High impact/negative life events (A)**
Number of negative life events; Impact of negative life events; Child witnessed a traumatic event; Child was a victim of a traumatic event
**High sexual involvement (A** **+** **D)**
Child had sexual intercourse multiple times with different persons; Child has an early sexual onset; Child doesn’t use birth control; Child is far in pubertal development; Child has children; Child has ever been pregnant or gotten someone pregnant
**History of grade retention (D)**
Child had a history of grade retention; Child is too old for grade level; Child repeated a grade
**Low academic achievement (A** **+** **D)**
Child had poor grades; Child has poor academic background; Child had a low Grade Point Average (GPA); Child is in a vocational high school program
**Low academic self-concept (A** **+** **D)**
Child expects upcoming grades to be bad; Child has a poor academic self-image
**Low IQ; learning difficulties (A** **+** **D)**
Child had low levels of general cognitive functioning; Child is in special education; Child had low scores on intelligence tests; Child has learning difficulties
**Negative or no leisure activities (A** **+** **D)**
Child is not participating in leisure time activities; Child is often loitering; Child doesn’t participate in any extracurricular activities; Child participated in passive activities, like watching TV
**Not being religious (A** **+** **D)**
Child is not, or only to a small extend, religious
**Other internalizing behavior (A** **+** **D)**
Child shows alienation; Child has internalizing problems; Child attempted or considered suicide; Child has a low self-esteem; Child has negative thoughts; Child has a panic disorder or symptoms; Child had somatic problems; Child is often tearful; Child is often withdrawn
**Poor physical health (A** **+** **D)**
Child is obese or overweight; Child is underweight; Child has a bad health; Child has a chronic illness; Child does not (or insufficiently) participate in physical exercise; Child has headaches; Child has migraine; Child has history of organic diseases; Child is impaired; Child has insomnia; Child has bad sleeping habits; Child has bad eating habits; Child has premenstrual symptoms; Child shows exhaustion
**Psychiatric symptoms; disorders(A** **+** **D)**
Child has a high total problem score on YRS; Child is autistic; Child is severely disables; Child is emotionally or behaviorally disabled; Child had psychiatric symptoms (in general)
**Risky coping/personality profile (A** **+** **D)**
Child is emotional instable; Child has an external locus of control; Child is extravert; Child is neurotic; Child is psychotic; Child is highly self-aware; Child is tough-minded; Child is closed; Child is pessimistic; Child is not agreeable; Child is not conscientious; Child shows low levels of self-efficacy; Child does not have a work drive; Child has personality problems; Child is repressive; Child uses non-problem solving coping, like avoidance and denial
**Showing risky behavior (A** **+** **D)**
Child drives without a license; Child drives when drinking alcohol; Child was involved in a traffic accident; Child drives in a not roadworthy vehicle; Child gets a real kick out of doing dangerous things; Child goes out at night beyond the neighborhood; Child does not wear a seatbelt; Child rides a motorbike; Child drives without a helmet; Child rode with a driver who had been drinking alcohol
**Smoking (A** **+** **D)**
Child is a (heavy) smoker; Child bought cigarettes; Child smokes cigars; Child started smoking early in life
**Family domains**
**Family structure (other than a nuclear family; A** **+** **D)**
Family breakup; Parental divorce; Child lives with a single parent/stepparents/in an institution/without parents;
**Having a history of child abuse victimization (A** **+** **D)**
Child is/was a victim of: child maltreatment/physical abuse/emotional abuse/physical neglect/sexual abuse; Child is/was a witness of domestic violence; Conflict within family; Spousal physical abuse
**Ineffective family systems and/or organization (e.g. low cohesion; A** **+** **D)**
Family disruptions or adversity; Low levels of cohesion/organization/expressiveness/intellectual-cultural orientation/moral-religious emphasis/achievement orientation within family/active-recreational orientation within the family; High levels of independency within the family
**Large family size (A** **+** **D)**
Large family size; High number of siblings within the family
**Low family SES (A** **+** **D)**
Low income of family members; Family lives in poverty; Child is homeless; Child receives free or reduced priced lunches at school; Child gets a low allowance from parents; Parents are unemployed; Child shared a room with siblings
**Low parental control and/or ineffective discipline (A** **+** **D)**
Low levels of monitoring, control or supervision by parents; There are no rules at home; Parents don’t offer structure; Parents punish children a lot; Parents use negative punishment; Lax or inconsistent parental discipline
**Low parental education (A** **+** **D)**
Low levels of parental education; Parents received no education; Parents were high school dropouts
**Low parental school involvement (A** **+** **D)**
Parents don’t help child with homework or other school stuff; Parents show low levels of communication with teachers or school; Parents have low expectations of a child’s school achievement; Parents don’t read with their child; Parents don’t support children with school related activities
**Low parental support/acceptance (A** **+** **D)**
Parents show high levels of rejection towards child; Parents don’t (or only to a small extent) encourage autonomy of child; Parent show low levels of acceptance towards child; Parents show low levels of involvement with child; Parents show low levels of affective support towards child; Parents show low levels of positive reinforcement towards child.
**Parental alcohol use (D)**
High levels of parental alcohol use
**Poor parent-child relationship (D)**
Low levels of parent-child communication/parent-child contact/parental sensitivity/attachment to parents/identification with parents
**Sibling at school (A)**
Sibling goes (used to go) to the same school
**Sibling dropped out (D)**
Sibling has dropped out of school
**School domains**
**Distance to school (short; A)**
Percentage of students living less than 1 mile from school
**Large classes; schools (A)**
Large classes; Large schools
**Low quality of school/education (A** **+** **D)**
Teacher doesn’t make it possible to participate in class; School has less advances math courses in school; Low achievement standards in school; Inadequate workload given to children by teacher; Poor quality of teachers (as perceived by children); Poor school management; Rapid instructional pace of teacher; Non-fair or non-effective school discipline methods; Poor school facilities; Low levels of commitment of school staff to school
**Negative school/class climate (A** **+** **D)**
Child feels unsafe at school; High levels of classroom competition; High levels of innovation in classroom; Child experiences ethnic, personal or sexual harassment in school; Rules within classroom are not clear; Low levels of order and organization within classroom; Low levels of task orientation within classroom; Low levels of school spirit
**Often changed schools (D)**
Family moved; Child attended different schools between kindergarten and 1th grade; Number of school changes; School moves
**Poor pupil-teacher relationship (A** **+** **D)**
Low levels of attachment to teacher; Low levels of commitment to teacher; High levels of control by teacher; Low teacher support; Negative attitudes toward teachers; Negative teacher attitudes towards student; Student-teacher conflict
**Public school (vs. private; A)**
School type (public = more risk)
**Peer domains**
**Being bullied (A)**
Child is victim of bullying; There is bullying within school; Child worries a lot about bullying
**Having a lot of friends/being popular (A** **+** **D)**
Child is accepted by peers; Child had a lot of friends; Child spends a lot of time with friends; High levels of identification with friends; Child is treated with respect by peers; Child is considered popular by peers
**Involvement with truant/deviant peers (D)**
Peers show low levels of school engagement; Deviant or dropped out peers; Child bonds with antisocial peers; Peers are truant
**Poor social competence (A** **+** **D)**
Child show poor social skills; Child shows low levels of social functioning; Child spends little time with friends; Child shows relational problems; Child shows poor social adjustment; Child is unpopular

*Note*. The risk domains are in boldface; A = School absenteeism; D = School dropout
